# Heat Transfer Enhancement in a Shell-and-Tube Type of Heat Exchanger Using Al_2_O_3_/Water Nanofluid

**DOI:** 10.1021/acsomega.6c03638

**Published:** 2026-09-17

**Authors:** Manuel Anthony Toribio Pacherres Uchalin, Edwin Santiago Rios Escalante, Luis Fernando Junior Saldaña Bernuy, Gustavo Henrique Romeu da Silva, Andrés Z. Mendiburu, Roberto C. Chucuya Huallpachoque, Alex M. Bimbato

**Affiliations:** † School of Engineering and Sciences, 28108 São Paulo State University (UNESP), Av. Dr. Ariberto Pereira da Cunha, 333, Guaratinguetá 12516-410, Brazil; ‡ Department of Mechanical Engineering, Federal University of Rio Grande do Sul (UFRGS), Porto Alegre Av. Paulo Gama, 110, Porto Alegre 90040-060, Brazil; § School of Energy Engineering, National University of Santa (UNS), Av. Universitaria S/N, Campus I, Nuevo Chimbote, Ancash 02711, Peru

## Abstract

Shell-and-tube heat exchangers are widely used in industrial processes, but improving the shell-side heat transfer without causing excessive pressure losses remains a major engineering challenge. Numerical simulations provide an efficient approach for evaluating thermohydraulic enhancement techniques while reducing experimental costs. The present study evaluates the heat transfer in a shell-and-tube heat exchanger using computational fluid dynamics by means of ANSYS Fluent software. Unlike previous numerical studies that mainly focused on thermal enhancement, this work provides a comprehensive thermohydraulic assessment by simultaneously evaluating heat-transfer improvement together with the associated pressure-drop and pumping-power penalties over different nanoparticle concentrations and mass flow rates. Therefore, a simplified heat-exchanger model was developed with six baffles operating under turbulent flow conditions. The working fluid in the shell is initially water, which is subsequently replaced by nanofluid composed of water and 15 nm Al_2_O_3_ nanoparticles, while the inner tube walls are kept at a constant temperature. The results show that the Al_2_O_3_/water nanofluid significantly enhances the heat transfer. Thermal conductivity and nanoparticle concentrations play a crucial role in this enhancement. The largest increase in heat transfer, 37.83%, occurs at a nanoparticle concentration of 1.25% at mass flow rates of 0.5 kg/s, 0.6 kg/s, and 0.7 kg/s. Lower concentrations result in 30.76% (concentration of 1.00%), 23.32% (concentration of 0.75%), 15.47% (concentration of 0.50%), and 7.16% (concentration of 0.25%) improvements compared to water. Similarly, the heat transfer coefficient increases by 47.84%, 38.32%, 28.62%, 18.70%, and 8.51%, for concentrations of 1.25%, 1.00%, 0.75%, 0.50%, and 0.25%, respectively. However, the use of nanofluids leads to an increase in the pressure drop, with maximum values reaching 5.30%. The results demonstrate that dilute Al_2_O_3_/water nanofluids can significantly enhance shell-side thermal performance while maintaining relatively low pressure drop and pumping power penalties, highlighting their potential as a practical thermo-hydraulic enhancement strategy for shell-and-tube heat exchangers and providing useful guidance for the design and optimization of industrial heat exchangers.

## Graphical abstract

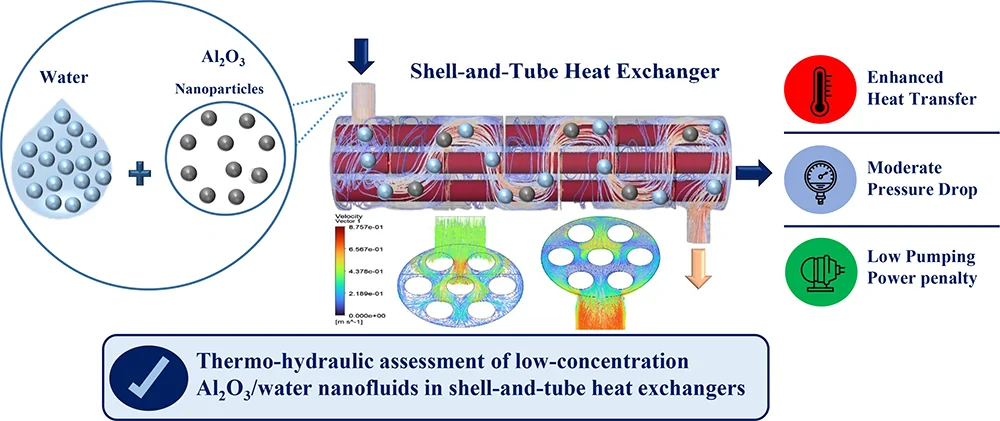

## 1 Introduction

In the past decade, increasing energy, the depletion of fossil fuels (such as oil, coal, and natural gas), climate change, atmospheric pollution, and the energy crisis have driven significant research on optimizing energy use to enhance efficiency and lower losses.^1,2^ One of the principal approaches involves improving heat-transfer technologies.^3^

Heat exchangers are essential components in energy transfer and conversion systems,^4^ widely used in industries such as oil and gas,^5^ manufacturing,^6^ petrochemicals,^7^ pharmaceuticals,^8^ HVAC,^9^ and refrigeration.^10^ Among them, the shell-and-tube heat exchanger is the most commonly used type.^11^

The shell-and-tube heat exchangers are commonly carried out in accordance with the standards established by the Tubular Exchanger Manufacturers Association (TEMA).^12^ For shell-side flow analysis, correlations such as the Kern method^13^ and Bell-Delaware approach^14^ are widely adopted due to their proven effectiveness.^15^ However, it is important to keep in mind that these correlations are based on physical principles, experimental data, and simplifications and therefore may not apply to all situations.^16^ For this reason, it is essential to validate the results obtained by using these correlations with specific experimental results. In the meantime, the use of numerical simulations can help to reduce the number of experimental tests, making experimental analyses less costly and time-consuming, which justifies the relevance of the study presented here.

According to Nakhchi and Esfahani,^17^ heat exchangers are essential devices for achieving energy savings in industrial processes. On the other hand, the thermophysical properties of the fluids commonly used in this equipment for heat transfer purposes are some of the fundamental factors that influence the heat exchange capacity of these fluids, which, in turn, are not always within the desired parameters.^18^ Therefore, given the constraints related to the dimensions, market value, and thermophysical properties of working fluids, it is essential to improve the performance of existing heat exchangers and to design new units that are lighter, more compact, and, consequently, less expensive.^19^

In order to overcome this condition and increase heat transfer, it is possible to change the boundary conditions, make geometric modifications to the equipment, or apply external agents.^20^ One of the most widespread methods for implementing the last alternative is to improve the thermophysical properties of the working fluid by adding nanoparticles. The result of this mixture is called a nanofluid.^21^

Nanofluids are composed of solid particles of metallic type, metal oxides, carbon-based nanomaterials such as carbon nanotubes, CNTs, or nonmetallic oxides, of nanometric size (less than 100 nm in diameter), which are dispersed in fluids used as coolants, such as water, ethylene glycol, propylene glycol, R600 refrigerant, or oil.^22^ This new fluid is used to improve the performance of thermal devices, such as heat exchangers,^21,23–26^ without altering their primary geometry.^27–29^ This use is justified by the low thermal conductivity of traditional refrigerants.^30^

The principal reason for this improvement in thermal energy exchange capacity is that this mixture has a combined effect of the properties of the base fluid and the nanoparticles.^31^ However, the effectiveness of nanofluids in promoting heat transfer can also be influenced by other factors, such as gravity, Brownian motion, Brownian diffusion, the friction force between the fluid and the nanoparticles, sedimentation, dispersion, and stratification at the solid/liquid interface; these factors may simultaneously interact within the main flow of this fluid.^18^ Consequently, several studies have focused on identifying the mechanisms that influence heat transfer in nanofluids while demonstrating that an improvement in thermal conductivity alone is not sufficient to explain the performance gains observed in the process.^32^

Nanofluids formulated with Al_2_O_3_ nanoparticles have attracted significant attention due to their superior thermal characteristics, high productivity, and stability.^33^ For instance, Main et al.^23^ investigated the thermal conductivity of trifluoromethylsulfonyl (as base fluid) and the thermal conductivity after the addition of Al_2_O_3_ nanoparticles at a 1.0% volumetric concentration, diameters of 10 nm, 30, 60, and 90 nm and varying temperatures (10 °C - 70 °C). The results showed that thermal conductivity increased by up to 14.63% when nanoparticles with a diameter of 10 nm were added at 70 °C.

Albadr et al.^34^ investigated the heat transfer performance of a shell-and-tube heat exchanger operating under countercurrent and turbulent flow conditions with Al_2_O_3_/distilled water nanofluids. Their findings indicated that dispersing nanoparticles into distilled water led to an enhancement in both the thermal conductivity and viscosity of the suspension, with the latter contributing significantly to the pressure drop. In addition, it was observed that the magnitudes of these effects increased with particle concentration. Moreover, when the nanoparticle concentration reached 2% by volume, the overall heat transfer coefficient improved by up to 57% compared to that of pure distilled water.

Another relevant contribution was presented by Oztürk, Tezcan, and Çiftçi,^35^ who performed a computational analysis on heat transfer enhancement in a shell-and-tube heat exchanger using Al_2_O_3_/water and CNT/water nanofluids at concentrations of 3%, 6%, and 9%. As expected, heat transfer increased with nanoparticle concentration. The maximum enhancement compared with pure water was approximately 50% and 62.5% at a concentration of 6% for Al_2_O_3_/water and CNT/water nanofluids, respectively. Although improvements were also observed at 9% concentration, the enhancement was reduced due to the dominant effect of viscosity at higher nanoparticle loadings, which negatively affects heat transfer, as reported in previous studies.^36^

Similarly, Gkountas et al.^37^ evaluated heat transfer enhancement in a supercritical CO_2_ Brayton cycle printed circuit heat exchanger using an Al_2_O_3_/water nanofluid. The results showed that a maximum volume fraction of 5% nanoparticles resulted in a 10% improvement in the heat-transfer coefficient compared to pure water. This improvement allowed for an additional 0.9% reduction in the length of the heat exchanger, along with a decrease in the pressure drop of up to 14%.

The Al_2_O_3_/water nanofluid has attracted the attention of the academic community and the industrial sector because of its innovative properties.^21^ According to the literature review, several studies have focused on evaluating the heat transfer enhancement in a shell-and-tube heat exchanger through the use of this nanofluid. These studies show that volumetric concentrations of Al_2_O_3_/water greater than 1% have been employed in this type of equipment, showing positive results in the heat transfer process but with higher pressure drop penalties. However, few studies address the use of Al_2_O_3_/water nanofluid circulating on the shell side of shell-and-tube heat exchanger at lower concentrations, as shown in Table 1.

**Table 1:** Summary of Previous Research on the Application of Al_2_O_3_/Water Nanofluid in Shell-and-Tube Heat Exchangers

Type of study	Nanoparticles concentration	Heat exchanger side	Reference
Numerical	1% and 2%	Tubes	^42^
Experimental	0.1%, 0.3% and 0.6%	Tubes	^43^
Numerical	1% and 5%	Tubes	^44^
Experimental	0.05%, 0.1% and 0.2%	Tubes	^45^
Numerical	0.01%, 0.03% and 0.05%	Tubes	^46^
Experimental and numerical	0.5%, 1%, 1.5% and 2%	Shell	^47^
Experimental	1%-3%	Tubes	^48^
Numerical	5%, 10%, 15% and 20%	Shell	^49^
Numerical	0.2%-2.5%	Tubes	^50^
Numerical	1%, 3% and 5%	Tubes	^51^
Experimental	0.02%, 0.1%, 0.2%, 0.4%, 0.6% and 0.8%	Tubes	^52^
Numerical	4%	Shell	^53^
Numerical	1%, 2%, 3% and 4%	Shell	^54^
Numerical	1%, 3% and 5%	Tubes	^55^
Numerical	0.5%-5%	Tubes	^56^
Numerical	1%, 3% and 5%	Tubes	^57^
Experimental	0.1%-8%	Tubes	^58^
Experimental	0.5%-4%	Tubes	^59^
Experimental	0.1%, 0.2% and 0.3%	Tubes	^60^
Numerical	1%, 2% and 3%	Shell	^61^
Experimental	0.5%, 1% and 1.5%	Tubes	^62^
Experimental	0.2%, 0.5% and 1%	Tubes	^63^
Numerical	0.3%, 0.5% and 0.75%	Tubes	^64^
Experimental	0.1%, 0.2% and 0.35%	Tubes	^65^
Experimental	0.2 y 0.4%	Tubes	^66^
Experimental	2% and 4%	Tubes	^67^
Numerical	0.3%, 0.5%, 0.75%, 1% and 2%	Tubes	^68^
Experimental and numerical	0.01%-0.1%	Tubes	^69^
Numerical	2%	Shell	^70^
Numerical	3%, 6% and 9%	Shell	^35^
Experimental and numerical	0.5%, 0.75%, 1.0%, 1.25% and 1.5%	Shell	^71^

Furthermore, although numerous studies have investigated nanofluids in heat exchangers, many have focused primarily on maximizing heat transfer enhancement without sufficiently addressing the thermohydraulic trade-off associated with increased pressure drop and pumping power requirements. Therefore, the present study focuses on evaluating the thermohydraulic performance of low-concentration Al_2_O_3_/water nanofluids under shell-side turbulent flow conditions, emphasizing the balance between heat transfer enhancement and hydraulic penalties in terms of pressure drop and pumping power requirements.

The novelty of the present work lies in the thermohydraulic evaluation of dilute Al_2_O_3_/water nanofluids with volume concentrations ranging from 0.25% to 1.25% under shell-side turbulent flow conditions in a shell-and-tube heat exchanger. Unlike many previous studies that primarily focused on heat transfer enhancement at higher nanoparticle concentrations, the present investigation jointly assesses thermal and hydraulic performance through the heat transfer rate, heat transfer coefficient, pressure drop, and pumping power. This approach provides practical design-oriented information regarding the feasibility of employing low-concentration nanofluids in industrial shell and tube heat exchangers.

For this purpose, a shell-and-tube heat exchanger operating with water and subsequently with Al_2_O_3_/water nanofluids at volumetric concentrations of 0.25%, 0.50%, 0.75%, 1.00%, and 1.25% was numerically investigated by using ANSYS Fluent 2024 R2. The sensitivity of the numerical results was assessed through a mesh independence study under water-only operating conditions. After an appropriate mesh was selected, simulations were conducted for three shell-side mass flow rates. The obtained results were then used to calculate the shell-side heat transfer coefficient and pressure drop and compared with the predictions of the Kern and Bell-Delaware correlations. Finally, the multiphase mixture model was employed to simulate the heat exchanger operating with a nanofluid.

Although several studies have investigated Al_2_O_3_/water nanofluids in heat exchangers, their practical implementation still requires detailed thermal-hydraulic evaluations under specific geometric and operating conditions. In this context, the novelty of the present work lies in the systematic CFD assessment of dilute Al_2_O_3_/water nanofluids in a shell-and-tube heat exchanger, considering not only heat-transfer enhancement but also pressure drop and pumping power. This combined analysis is relevant because the use of nanofluids is only advantageous when excessive hydraulic penalties do not offset the heat transfer improvement. Therefore, the present study contributes to identifying operating conditions under which dilute Al_2_O_3_/water nanofluids may provide a favorable compromise between thermal performance and pumping power requirements.

Recent studies have also demonstrated that passive geometric strategies, including twisted, corrugated, conical, and multihelical tube configurations, can intensify secondary flows and fluid mixing, thereby improving heat transfer and overall thermo-hydraulic performance. However, these thermal gains should be evaluated together with the resulting frictional losses, pressure drop, pumping requirements, and exergy behavior to determine whether the enhancement provides a favorable engineering compromise.^38–41^

## 2 Methodology

In computational fluid dynamics (CFD), the analysis of a system generally consists of three important stages: preprocessing, processing, and postprocessing. Figure 1 shows the workflow of the CFD methodology adopted in this study, beginning with the preprocessing stage, which involves the creation, generation, and naming selections of the heat exchanger domain, as well as its respective mesh. Next comes the processing stage, in which the materials are specified, boundary conditions are imposed, and governing equations are solved. Finally, the postprocessing stage, during which the results calculated by the software are obtained, is followed by an overview of the flow behavior. It is important to note that this three-step process is repeated, even under different conditions, in order to obtain a set of results that can be analyzed and compared.^68,72^

**Figure 1:**
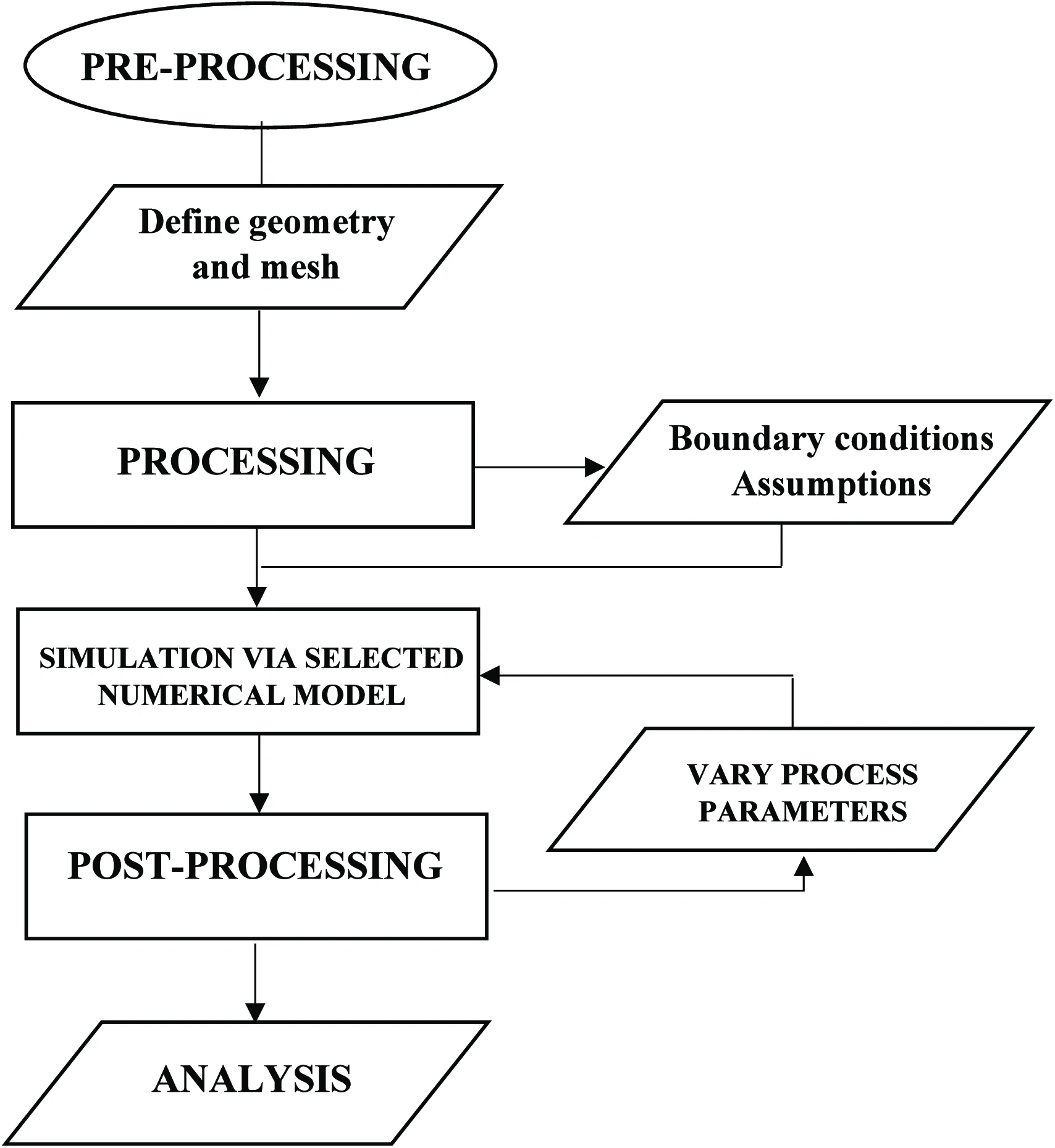
Stages of a CFD process. Adapted with permission from ref 68.

The software used to carry out this process was ANSYS 2024 R2, executed on a dual liquid-cooled CPU computer with 12 cores, 24 threads, 64GB of memory, and a 3200 MHz frequency.

The commercial CFD software ANSYS Fluent was employed as the numerical solver to assess improvements in heat transfer and to quantify the associated pressure drop when using an Al_2_O_3_/water nanofluid as a coolant in a heat exchanger under turbulent-flow conditions. To validate the general CFD modeling strategy without nanofluids, a previous study by Uchalin,^73^ based on the work of Ozden and Tari,^74^ demonstrated the reliability of a comprehensive CFD representation of a laminar-flow heat exchanger by benchmarking the predictions against experimental measurements obtained from a small-scale educational unit containing two baffles. Similarly, to verify and support the general approach to CFD modeling including nanofluids, a previous study by Uchalin (2024),^73^ based on the work of Bianco et al.,^75^ demonstrated the accuracy of a CFD model for a circular tube in a turbulent regime using nanofluids by correlating the outcomes with experimental data reported in the literature.

Although these validation studies provide confidence in the adopted numerical methodology, direct experimental measurements for the specific shell-and-tube heat exchanger configuration analyzed in the present work were not available. Therefore, the comparisons with the Kern and Bell–Delaware methods should be interpreted as a benchmarking and verification procedure rather than as a substitute for direct experimental validation.

### 2.1 Pre-Processing

#### 2.1.1 Geometry

The simplified geometry of the heat exchanger, adapted from Ozden and Tari^74^ and illustrated in Figure 2, corresponds to a pilot-scale configuration in which the thicknesses of the tubes and the shell are neglected. In addition, it excludes design aspects such as flanges, supports, and other features that are not of interest for the CFD analysis since including them would not influence the results and would only increase the cost in terms of computational time. The three-dimensional model was developed by using the ANSYS SpaceClaim CAD tool. The technical specifications of the shell-and-tube heat exchanger are summarized in Table 2.^12^

**Figure 2:**
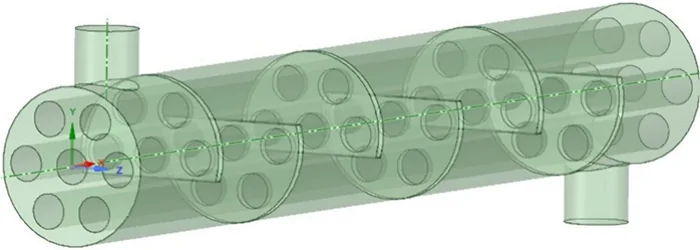
Geometry of a shell-and-tube heat exchanger at pilot scale.

**Table 2:** Technical Specifications of the Shell-and-Tube Heat Exchanger

Description	Specification	Reference
Heat exchanger length	600 mm	^12^
Shell outside diameter	90 mm	
Tube outside diameter	20 mm	
Geometry and distribution of the tube bundle	Triangular, 30 mm	
Number of tubes	7	
Baffle cut	36%	
Number of baffles	6	
Central baffle spacing	86 mm	
Nozzle diameter	36 mm	

#### 2.1.2 Meshing and Mesh Independence Test

The mesh generation was carried out using Fluent meshing. The mesh elements generated for the entire computational domain are of the unstructured hybrid type, as the automatic polyhedral meshing method was applied. Regarding the mesh structure, the shell-side fluid domain was discretized using polyhedral cells to improve cell connectivity and reduce the total number of elements, thereby optimizing computational efficiency. Since this is a conjugate heat transfer problem (solid–fluid interaction), local mesh refinement was applied around the tubes and baffle surfaces, where strong velocity and temperature gradients are expected. In addition, prismatic boundary-layer elements were generated adjacent to the tube walls and baffle surfaces to improve the resolution of the near-wall flow and thermal fields. Although a target *y* ^*+*^ value was not prescribed *a priori* during mesh generation, prismatic boundary-layer elements were introduced near the tube walls and baffles to improve the resolution of near-wall velocity and temperature gradients. The adopted near-wall refinement, combined with the mesh-independence analysis and the agreement obtained with the Bell–Delaware method, indicates that the selected mesh provides a reasonable compromise between numerical accuracy and computational cost. Nevertheless, a dedicated *y* ^*+*^-based wall-treatment sensitivity analysis was not performed and is acknowledged as a limitation of the present study. Details of the resulting mesh are presented in Figure 3.

**Figure 3:**
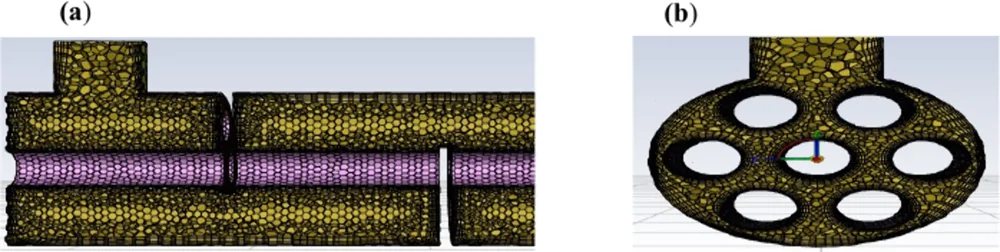
Mesh representation of the shell-and-tube heat exchanger: a) longitudinal section and (b) cross-sectional view.

To ensure the adequacy of the applied mesh, we conducted a mesh independence test. This test constitutes a fundamental step in the validation of numerical simulations, particularly in heat transfer applications.^3,76–80^ The use of an overly coarse mesh may result in the loss of important flow and heat-transfer physics, potentially leading to the underestimation or overestimation of temperature gradients and heat-transfer rates. Conversely, an excessively refined mesh can cause an unnecessary increase in the computational time without yielding a significant improvement in the accuracy of the results.

In this context, Table 3 presents a comparison between the heat transfer rates obtained through numerical simulations and those derived from analytical methods, when the heat exchanger operates solely with water. As part of the mesh independence analysis, three levels of mesh refinement are analyzed: a coarse mesh composed of 250,566 elements, a medium mesh composed of 813,116 elements, and a fine mesh composed of 1,569,090 elements. The fine mesh was ultimately selected for the numerical simulations, as the number of elements compared to the coarser meshes did not entail a significant increase in computational cost, while offering improved accuracy in the results.

**Table 3:** Results Obtained during the Mesh Independence Test

					Analytical calculations	
				Results of the CFD analysis	Bell-Delaware method	
Case	Viscous model	Mesh	Mass flow rate (kg/s)	Total heat transfer rate (kW)	Total heat transfer rate (kW)	Error (%)
A	κ - *ε* Realizable second order	Coarse mesh	0.5	84.43	81.39	3.74
			0.6	97.49	95.46	2.13
			0.7	111.79	109.76	1.85
B		Medium mesh	0.5	86.06	84.03	2.42
			0.6	99.68	98.65	1.04
			0.7	115.15	113.10	1.81
C		Fine mesh	0.5	85.04	84.99	0.06
			0.6	99.96	99.91	0.05
			0.7	114.98	114.91	0.06

### 2.2 Processing

#### 2.2.1 Governing Equations

The physical phenomena addressed in this study are governed by the fundamental conservation laws of mass (eqs 1), linear momentum (eqs 2), and energy (eqs 3). These equations, which represent the physical principles of conservation, are employed to evaluate the velocity, pressure, and temperature fields arising from the motion of a conventional fluid within an arbitrary geometry.^81^ In this work, the governing equations are expressed using indicial notation, as follows:

1 $$\frac{\partial \rho }{\partial t}+\frac{\partial }{\partial x_{i}}\left(\rho u_{i}\right)=0$$

2 $$\frac{\partial \left(\rho u_{i}\right)}{\partial t}+\frac{\partial }{\partial x_{j}}\left(\rho u_{j}u_{i}\right)=-\frac{\partial p}{\partial x_{i}}+\frac{\partial }{\partial x_{j}}\left(\mu \frac{\partial u_{i}}{\partial x_{j}}\right)+\rho g_{i}-\frac{2}{3}\frac{\partial }{\partial x_{i}}\left(\mu \frac{\partial u_{j}}{\partial x_{j}}\right)+\frac{\partial }{\partial x_{j}}\left(\mu \frac{\partial u_{j}}{\partial x_{i}}\right)$$

3 $$\frac{\partial \left(\rho T\right)}{\partial t}+\frac{\partial }{\partial x_{j}}\left(\rho u_{j}T\right)=\frac{\partial }{\partial x_{j}}\left(\frac{k}{c_{p}}\frac{\partial T}{\partial x_{j}}\right)+\frac{1}{c_{p}}\frac{Dp}{Dt}+\frac{\mu }{c_{p}}\Phi$$

where ρ denotes the fluid density, *u*_*i*_ represents the *i* component of the velocity vector, and *u*_*j*_ corresponds to the *j* component of the fluid velocity vector. The variable *p* refers to the pressure field, while μ is the dynamic viscosity of the fluid. The term *g*_*i*_ denotes the *i* component of the local gravitational acceleration vector. Additionally, *T* is the temperature field, *k* is the thermal conductivity of the fluid, *c*_*p*_ represents the specific heat at constant pressure, and Φ is the viscous dissipation function.

It is worth noting that the equations (eqs 1–3) are integrated into the ANSYS Fluent solver for performing the simulations. These equations are solved in simplified form according to the assumptions adopted in the present study. Furthermore, the cooling fluid is assumed to be incompressible (except in the buoyancy term, where the Boussinesq approximation is used) and in steady-state conditions; that is, the fluid’s properties may vary with position but not with time. A steady-state approach was adopted because the present study focuses on evaluating the average performance of the heat exchanger under constant operating conditions. In all simulations, the inlet mass flow rate, inlet temperature, and wall temperature were maintained constant, with no transient thermal or hydrodynamic disturbances imposed on the system. In addition, the following assumptions are adopted for the case analyzed in this study:

- The nanofluid is considered homogeneous; according to Garoosi et al.,^82^ this is valid for nanoparticle volume concentrations below 5%.
- The nanofluid is modeled as a Newtonian fluid; as indicated by Chen et al.,^83^ this approximation is appropriate for nanoparticle concentrations under 5%.
- The thermophysical properties of both the base fluid and the nanoparticles are assumed to be constant, uniform, and isotropic.
- The base fluid and the nanoparticles are assumed to be in hydrodynamic equilibrium.
- Mass and interfacial motion are neglected.
- Nondrag interfacial forces, such as lift forces, wall lubrication effects, and virtual mass, are disregarded.

Furthermore, this study focused exclusively on the influence of nanoparticle concentration on the performance of the shell and tube heat exchanger. Other important factors affecting nanofluid behavior, such as nanoparticle size, dispersion stability, sedimentation, and agglomeration effects, were not explicitly modeled or analyzed. For instance, smaller nanoparticles may enhance the thermal properties of nanofluids and consequently improve heat transfer.^24^ In contrast, larger nanoparticles, higher pressure drops, and increased pumping power represent important challenges for future research.^84,85^

However, the investigated concentration range was limited to dilute conditions to minimize excessive agglomeration and stability-related effects commonly observed at higher nanoparticle concentrations.

It should be noted that the mixture model adopted in this study provides a computationally efficient framework for simulating dilute nanofluid suspensions. Nevertheless, this approach assumes local equilibrium between the base fluid and nanoparticles and does not explicitly capture complex phenomena such as particle agglomeration, sedimentation dynamics, or other concentration-dependent interactions that may become significant at higher nanoparticle loadings. Consequently, the present results should be interpreted within the scope of dilute and relatively stable nanofluid suspensions.

#### 2.2.2 Turbulence Model and Multiphase Model

At present, there is no universally accepted complete theory of turbulence. However, over the past half-century, following the pioneering theoretical contributions of Prandtl, Kolmogorov, and Von Karman,^86^ numerous theoretical and computational strategies have been developed to characterize this phenomenon. These strategies are employed depending on the problem being addressed and the computational resources available. Among the existing numerical approaches for solving turbulent flows are the Direct Numerical Simulation (DNS), the Large Eddy Simulation (LES), and Reynolds Averaged Navier–Stokes Equations (RANS), the latter of which is the strategy adopted in this study. To close the turbulence problem, it is necessary to compute the turbulent viscosity (μ_*t*_).^87^ In the present study, this parameter is calculated using the κ - *ε* realizable two-equation model developed by Shih et al.,^88^ where κ represents the turbulent kinetic energy and *ε* the rate of dissipation of turbulent kinetic energy.^89^ This model was selected due to its accuracy in preliminary tests, good convergence behavior, and relatively low memory requirements, making it a suitable alternative for simulating turbulent flows and heat transfer.^90^ It should be noted that comparative tests were conducted using different turbulence models, including the standard *k*–*ε* and Spalart–Allmaras models, as well as first- and second-order discretization schemes and three mesh refinement levels. Cases employing the standard *k*–*ε* and Spalart–Allmaras models were discarded because they produced physically inconsistent results. In some cases, the shell-side outlet temperature increased with increasing mass flow rate, leading to unacceptable predictions of the total heat transfer rate. In other cases, although the outlet temperature trend was physically consistent, the deviation in the predicted total heat transfer rate relative to the Bell–Delaware method increased as the mass flow rate decreased. These discrepancies were even more pronounced when compared with the Kern method results, as previously discussed in this study.

In the realizable κ-ε model, turbulent viscosity is obtained from the turbulent kinetic energy and its dissipation rate, according to eqs 4 and ([Disp-formula eq5]).^87^

4 $$\frac{\partial \kappa }{\partial t}+{\mathrm{u̅}}_{j}\frac{\partial \kappa }{\partial x_{j}}=\frac{\tau_{ij}^{’}}{\rho }\frac{\partial {\mathrm{u̅}}_{i}}{\partial x_{j}}-\varepsilon +\frac{\partial }{\partial x_{j}}\left[\left(\frac{\mu }{\rho }+\frac{\mu_{t}}{\rho \sigma_{\kappa }}\right)\frac{\partial \kappa }{\partial x_{j}}\right]$$

5 $$\frac{\partial \varepsilon }{\partial t}+{\mathrm{u̅}}_{j}\frac{\partial \varepsilon }{\partial x_{j}}=C_{\varepsilon_{1}}\frac{\varepsilon }{\kappa }\frac{\tau_{ij}^{’}}{\rho }\frac{\partial {\mathrm{u̅}}_{i}}{\partial x_{j}}+C_{2}\frac{\varepsilon^{2}}{k+\sqrt{\nu \varepsilon }}+C_{1}+\frac{\partial }{\partial x_{j}}\left[\left(\frac{\mu }{\rho }+\frac{\mu_{t}}{\rho \sigma_{\varepsilon }}\right)\frac{\partial \varepsilon }{\partial x_{j}}\right]$$

Where τ_*ij*_^′^ is the Reynolds stress tensor, and *u̅*_*i*_ and *u̅*_*j*_ are the average components of the velocity vector (Reynolds decomposition typical of the RANS technique). The coefficients of the equations used in this model are listed in Table 4.^91^

**Table 4:** Detailed Forms of the Coefficients in eqs 4 and ([Disp-formula eq5])

Coefficient	Value or equation for calculation	Physical significance
τ_*ij*_^′^	$\rho \overset{®}{u_{i}^{’}u_{j}^{’}\;}=\mu_{t}\left(\frac{\partial {\mathrm{u̅}}_{i}}{\partial x_{j}}+\frac{\partial {\mathrm{u̅}}_{j}}{\partial x_{i}}\right)$	Reynolds stress tensor
μ_*t*_	$\rho C_{\mu }\frac{\kappa^{2}}{\varepsilon }$	Turbulent viscosity
*C*_1_	$\max\left[0.43,\frac{\eta }{\eta +5}\right]$	Model constant
*C*_2_	1.90	Model constant
*C*_*ε*_1__	1.44	Model constant
η	$\sqrt{2S_{ij}S_{ij}}\frac{\kappa }{\varepsilon }$	Ratio of turbulent time scale to the time-averaged strain rate
*S*_*ij*_	$\frac{1}{2}\left(\frac{\partial {\mathrm{u̅}}_{j}}{\partial x_{i}}+\frac{\partial {\mathrm{u̅}}_{i}}{\partial x_{j}}\right)$	Time-averaged strain rate of the main flow

In this work, the pressure–velocity coupled scheme was applied for solving the governing equations, a method widely recognized for its robustness and efficiency in steady-state simulations.^77^ Preliminary evaluations confirmed that it outperformed the segregated approach, which justified its adoption in this study. Regarding spatial discretization, a second order upwind scheme was employed for both convective and diffusive terms acting on pressure in a single-phase case, whereas the PRESTO scheme was adopted when simulating nanofluid flows with the multiphase model. Second-order schemes were also adopted for momentum, energy, turbulent kinetic energy, and turbulent energy dissipation rate. In contrast, the volume fraction of the secondary phase was discretized using a first-order upwind scheme. These discretization schemes were chosen due to their simplicity, rapid convergence, improved accuracy, and lower susceptibility to numerical oscillations.^92^ Since there is currently no universally accepted convergence criterion for numerical flow simulations,^93^ residual monitoring is commonly adopted as a reliable indicator during the iterative solution process. In the present study, convergence criteria of 10^–3^ were imposed for the continuity, momentum, turbulent kinetic energy, turbulent dissipation rate, and nanoparticle volume fraction equations (when operating with nanofluid), while a stricter criterion of 10^–6^ was applied to the energy equation.

In addition to residual monitoring, the convergence of the simulations was verified through the stabilization of global thermohydraulic parameters, including the shell-side outlet temperature, total heat transfer rate, and pressure drop, which exhibited negligible variation with further iterations.

It is important to make it clear that, from this point onward in the text, for typing convenience, the bar that designates average property values will be omitted.

In accordance with the considerations presented in section 2.2, and based on the criteria established in a previous study,^73^ regarding the appropriate selection of the multiphase model, the mixture model was chosen for this work. This model is the simplest approach to analyzing two-phase flows. In this model, the conservation equations for mass, momentum, and energy, along with the volumetric concentration equation of the secondary phase, are solved. Consequently, the relative velocity between the phases is obtained using algebraic expressions.^94^ The governing equations of this model have been widely addressed in numerical studies and are presented below.^95–97^

Mass:

6 $$\nabla ⃗\cdot \left(\rho_{m}{\mathrm{u⃗}}_{m}\right)=0$$

Momentum:

7 $$\nabla ⃗\cdot \left(\rho_{m}{\mathrm{u⃗}}_{m}{\mathrm{u⃗}}_{m}\right)=-\nabla ⃗P_{m}+\nabla ⃗\cdot \tau_{m}+\rho_{m}\mathrm{g⃗}-\nabla ⃗\cdot \overset{2}{\underset{k=1}{\sum }}\varphi_{k}\rho_{k}{\mathrm{u⃗}}_{dr,k}{\mathrm{u⃗}}_{dr,k}$$

Energy:

8 $$\nabla ⃗\cdot \left(\rho_{m}{\mathrm{u⃗}}_{m}H_{m}\right)=-\nabla ⃗\cdot \left(q_{m}+q_{t}\right)-\nabla ⃗\cdot \overset{2}{\underset{k=1}{\sum }}\varphi_{k}\rho_{k}{\mathrm{u⃗}}_{dr.k}H_{m}+\nabla ⃗\cdot \overset{2}{\underset{k=1}{\sum }}\varphi_{k}\tau_{k}\cdot \nabla ⃗{\mathrm{u⃗}}_{m}$$

Volumetric concentration equation:

9 $$\nabla ⃗\cdot \left(\varphi_{p}{\mathrm{u⃗}}_{m}\right)=-\nabla ⃗\cdot \left(\varphi_{p}{\mathrm{u⃗}}_{dr,p}\right)$$

the subscript *m* refers to the mixture, and the subscript *k* denotes the phase *k*, where ρ_*k*_ is the density of phase *k*, φ_*k*_ is the volume fraction of phase *k*, and *H*_*m*_ represents the enthalpy of the mixture. The term *q*_*m*_ + *q*_*t*_ corresponds to an increment in energy diffusion due to turbulence. According to previous studies,^79,98,99^ the relevant parameters are calculated as follows:

Mixture density:

10 $$\rho_{m}=\overset{n}{\underset{k=1}{\sum }}\varphi_{k}\rho_{k}$$

Mixture velocity:

11 $${\mathrm{u⃗}}_{m}=\frac{\overset{n}{\underset{k=1}{\sum }}\varphi_{k}\rho_{k}{\mathrm{u⃗}}_{k}}{\rho_{m}}$$

Mixture viscous tensor:

12 $$\tau_{m}=\nabla ⃗\cdot [\left(\mu_{m}+\mu_{t}\right)\left(\nabla ⃗{\mathrm{u⃗}}_{m}+\nabla ⃗{\mathrm{u⃗}}_{m}^{T}\right]$$

where the term μ_*m*_ + μ_*t*_ represents an increase in momentum diffusion due to turbulence, analogous to the term *q*_*m*_ + *q*_*t*_ in the energy equation (eq 8).

In the momentum equation, the drift velocity in the secondary phase (nanoparticles) is defined as follows:

13 $${\mathrm{u⃗}}_{dr,k}={\mathrm{u⃗}}_{k}-{\mathrm{u⃗}}_{m}$$

The relative velocity (also referred to as slip velocity), denoted as *u⃗*_*pq*_, is defined as the velocity of the secondary phase with respect to the primary phase in a multiphase system. It can be calculated using the following equations:

14 $${\mathrm{u⃗}}_{pq}={\mathrm{u⃗}}_{p}-{\mathrm{u⃗}}_{q}$$

15 $${\mathrm{u⃗}}_{pq}=\frac{\rho_{p}d_{p}^{2}}{18\mu_{q}f_{drag}}\frac{\left(\rho_{p}-\rho_{m}\right)}{\rho_{p}}\left[\mathrm{g⃗}-\left({\mathrm{u⃗}}_{m}\cdot \nabla ⃗\right){\mathrm{u⃗}}_{m}-\frac{\partial {\mathrm{u⃗}}_{m}}{\partial t}\right]$$

in eqs 14 and ([Disp-formula eq15]), the subscripts *p* and *q* refer to the nanoparticles (secondary phase) and the base fluid (primary phase), respectively, where *u⃗*_*q*_ is the velocity of the base fluid, *u⃗*_*p*_ is the velocity of the nanoparticles, and *f*_*drag*_ is the drag function. The latter is given by^100^

16 fdrag={1+0.15Re0,687⁣Re≤1.0×1030.0183Re⁣Re>1.0×103}

17 Re=dpρqu⃗pqμq

where *Re* is the relative Reynolds number, and *d*_*p*_, ρ_*q*_, and μ_*q*_ represent the diameter of the nanoparticle, the density of the base fluid, and the dynamic viscosity of the base fluid, respectively.

The drift velocity and the relative velocity (*u⃗*_*pq*_) are related by the following expression:

18 $${\mathrm{u⃗}}_{dr,p}={\mathrm{u⃗}}_{pq}-\overset{n}{\underset{k=1}{\sum }}\frac{\varphi_{k}\rho_{k}}{\rho_{m}}{\mathrm{u⃗}}_{kq}$$

where ρ_*k*_ is the density of the nanoparticles, and *u⃗*_*kq*_ is the velocity of the phase *k* relative to phase *q*.

Although the mixture model has been widely employed for nanofluid simulations because of its relatively low computational cost, its applicability is generally more appropriate for dilute nanoparticle suspensions. Previous studies have shown that the model provides satisfactory predictions at low nanoparticle concentrations, whereas its accuracy may deteriorate as the nanoparticle loading increases.^75,101,102^ This limitation arises because the mixture model primarily accounts for drag interactions between the continuous and dispersed phases and does not explicitly consider particle–particle interactions, agglomeration phenomena, or collision effects, which may become increasingly important at higher nanoparticle concentrations. Since the present study is restricted to Al_2_O_3_/water nanofluids with volume concentrations of up to 1.25%, the mixture model is considered an appropriate compromise between computational efficiency and predictive capability.

#### 2.2.3 Fluid Properties

The thermophysical properties of the base fluid were defined in the software as temperature-independent. The properties of the nanofluid (Al_2_O_3_/water) were modeled as those of a homogeneous fluid by specifying its effective parameters. The dispersed Al_2_O_3_ nanoparticles were considered with a mean diameter of 15 nm, and according to Gupta et al.,^32^ a density of 3970 kg/m^3^, a specific heat of 0.765 kJ/kg.K, and a thermal conductivity of 36 W/m·K. In nanofluids analysis, the most important parameters are thermal conductivity, density, specific heat, and dynamic viscosity. However, to calculate these properties, it is first necessary to determine the volumetric concentration of the nanoparticles (eq 19).^103^ Once this value is known, the relevant thermophysical properties can be obtained using eqs 20 to ([Disp-formula eq23]).^104–106^ The thermophysical properties used in the calculations were obtained from Incropera et al.^107^

19 $$\varphi_{np}=\frac{\left(\frac{m_{np}}{\rho_{np}}\right)}{\left(\frac{m_{np}}{\rho_{np}}\right)+\left(\frac{m_{fb}}{\rho_{fb}}\right)}$$

20 $$k_{nf}=k_{fb}\times \frac{k_{np}+2k_{fb}+2\varphi_{np}\left(k_{np}-k_{fb}\right)}{k_{np}+2k_{fb}-\varphi_{np}\left(k_{np}-k_{fb}\right)}$$

21 $$\rho_{nf}=\varphi_{np}\times \rho_{np}+\left(1-\varphi_{\mathrm{np}}\right)\rho_{fb}$$

22 $$\mu_{nf}=\frac{\mu_{fb}}{{\left(1-\varphi_{np}\right)}^{2,5}}$$

23 $$c_{p,nf}=\frac{\left(\varphi_{np}\right)\rho_{np}c_{p,np}+\left(1-\varphi_{np}\right)\rho_{fb}c_{p,fb}}{\rho_{nf}}$$

where *m* denotes mass, *k* denotes thermal conductivity, ρ denotes density, μ denotes dynamic viscosity, and *c*_*p*_ denotes specific heat. The subscripts *np*, *fb*, and *nf* refer to nanoparticles, base fluid and nanofluid, respectively.

The percentage difference obtained from the comparison between the thermophysical properties of the base fluid and those of the nanofluid is detailed in the work of Uchalin et al.^73^ This information supports the results obtained for parameters such as the heat transfer rate, overall heat transfer coefficient, and pressure drop, which will be presented in subsequent sections.

#### 2.2.4 Boundary Conditions

On the shell side, the fluid used was first water and later replaced with the nanofluid, both entering through a mass flow inlet boundary condition at a fixed temperature of 27 °C. An adiabatic condition was imposed on the outer surface of the shell. On the tube side, the walls were maintained at a uniform temperature of 177 °C. A no-slip boundary was enforced over every solid surface, and a pressure outlet boundary condition with a reference pressure of zero gauge was specified at the outlet to quantify the pressure difference between inlet and outlet. The study is conducted under three variations of mass flow rate (0.5, 0.6, and 0.7 kg/s) and five variations of Al_2_O_3_ nanoparticles volume fraction (0.25%, 0.50%, 0.75%, 1.00%, and 1.25%).

#### 2.2.5 Pumping Power

According to the pumping power equation, the variation of pumping power is associated with the mass flow rate, pressure drop, and density of working fluids.^108^ The pumping power (*P*_*p*_) of the nanofluids can be calculated as^109^

24 $$P_{pp}=\left(\frac{{ṁ}_{nf}}{\rho_{nf}}\right)\times \Delta p_{nf}$$

Here, Δ*p* is the pressure drop (Pa), *ṁ* is the mass flow rate, and ρ is the density of nanofluid.

### 2.3 Post-Processing

Postprocessing allows the retrieval of relevant outcomes from the simulations. After the solution converges, ANSYS Fluent provides detailed reports on flow properties and surface-related quantities. Key outputs include the heat transfer rate, the outlet temperature of the fluid on the shell side, and the associated pressure drop, which are obtained directly through the solver. In this study, the heat transfer coefficient, *h*, was calculated using eqs 25 and 26.^110^

25 $$U_{o}=\frac{\mathrm{Q̇}}{A_{o}\Delta T_{ml}}$$

26 $$A_{o}=N_{t}\pi d_{o}L_{t}$$

where *Q̇* is the total heat transfer rate, Δ*T*_*ml*_ is the logarithmic mean temperature difference, *A*_*o*_ is the heat transfer area of the tubes, *N*_*t*_ is the number of tubes, *d*_*o*_ is the tube diameter, and *L*_*t*_ the tube length.

Since the heat exchanger was simplified and the internal tube walls were assumed to be at a constant temperature, the external convective heat transfer coefficient, *h*, was considered equal to overall heat transfer coefficient, *U*_0_, due to the negligible internal thermal resistance.

In addition to these postprocessing parameters, ANSYS Fluent also provides an assessment of the overall flow behavior through velocity, pressure, and temperature contours, which facilitates the evaluation of the nanofluid’s performance as a coolant in the heat exchanger.

## 3 Results and Discussions

### 3.1 CFD Model Verification and Benchmarking

This section presents the results obtained for heat transfer in a shell-and-tube heat exchanger operating with water in a turbulent flow regime. Table 5 shows the results obtained through numerical simulations in ANSYS Fluent. These results are compared with those obtained from analytical correlations (see Table 6) to evaluate the model’s ability to represent the thermal performance of heat exchanger. It is important to highlight that all the results were obtained using the most refined mesh. Additionally, the percentage differences between the numerical and analytical results are summarized in Table 7.

**Table 5:** Results of Numerical Analysis

Mesh refinement	Turbulence model	Mass flow rate (kg/s)	Outlet temperature (°C)	Heat transfer coefficient (kW/m^2^ °C)	Pressure drop (kPa)	Heat transfer rate (kW)
Fine mesh 1,569,090 elements	κ - *ε* Realizable second order	0.5	67.66	2.505	1.242	85.04
		0.6	66.83	2.934	1.806	99.96
		0.7	66.27	3.367	2.462	114.98

**Table 6:** Analytical Calculation Results

	Kern method		Bell-Delaware method	
Mass flow rate (kg/s)	Heat transfer coefficient (kW/m^2^·°C)	Heat transfer coefficient (kW/m^2^·°C)	Pressure drop (kPa)	Heat transfer rate (kW)
0.5	2.291	2.595	1.221	84.99
0.6	2.546	2.891	1.785	99.91
0.7	2.769	3.174	2.384	114.91

**Table 7:** Percentage Difference between the Results of the Numerical Analysis and the Analytical Calculations

	Kern method		Bell-Delaware method	
Mass flow rate (kg/s)	Heat transfer coefficient	Heat transfer coefficient	Pressure drop	Heat transfer rate
0.5	9.3%	3.5%	1.7%	0.06%
0.6	15.2%	1.5%	1.2%	0.05%
0.7	21.6%	6.1%	3.3%	0.06%

Table 5 shows that as the mass flow rate increases, the outlet temperature decreases. This occurs because a higher mass flow rate of water implies a greater heat storage capacity, while at the same time, there is an increase in the heat transfer coefficient and, consequently, an increase in the heat transfer rate from the tube surface to the water. However, as the mass flow rate increases, the pressure drop increases. This is due to the turbulence caused by the geometry of the heat exchanger considered in this study (see Section 2.1)

Table 7 indicates that the percentage deviations obtained when contrasting the numerical simulations outcomes with the analytical correlations, for both heat transfer rate and pressure drop, remain within acceptable ranges. The same applies to the heat transfer coefficients for all mass flow rates, considering only the Bell-Delaware method in this comparison, as it offers greater accuracy. It is worth mentioning that, for the development of the numerical model, a sensitivity analysis was performed using different turbulence models to accurately capture the physical phenomena involved in turbulent flows. In this analysis, the realizable κ-ε model demonstrated superior performance in predicting flow patterns and heat transfer, providing results in closer agreement with the analytical calculations.

### 3.2 Heat Transfer Rate of Al_2_O_3_/Water Nanofluid

Figure 4 shows the variation in the heat transfer rate as a function of mass flow rates for five different concentrations of nanofluids.

**Figure 4:**
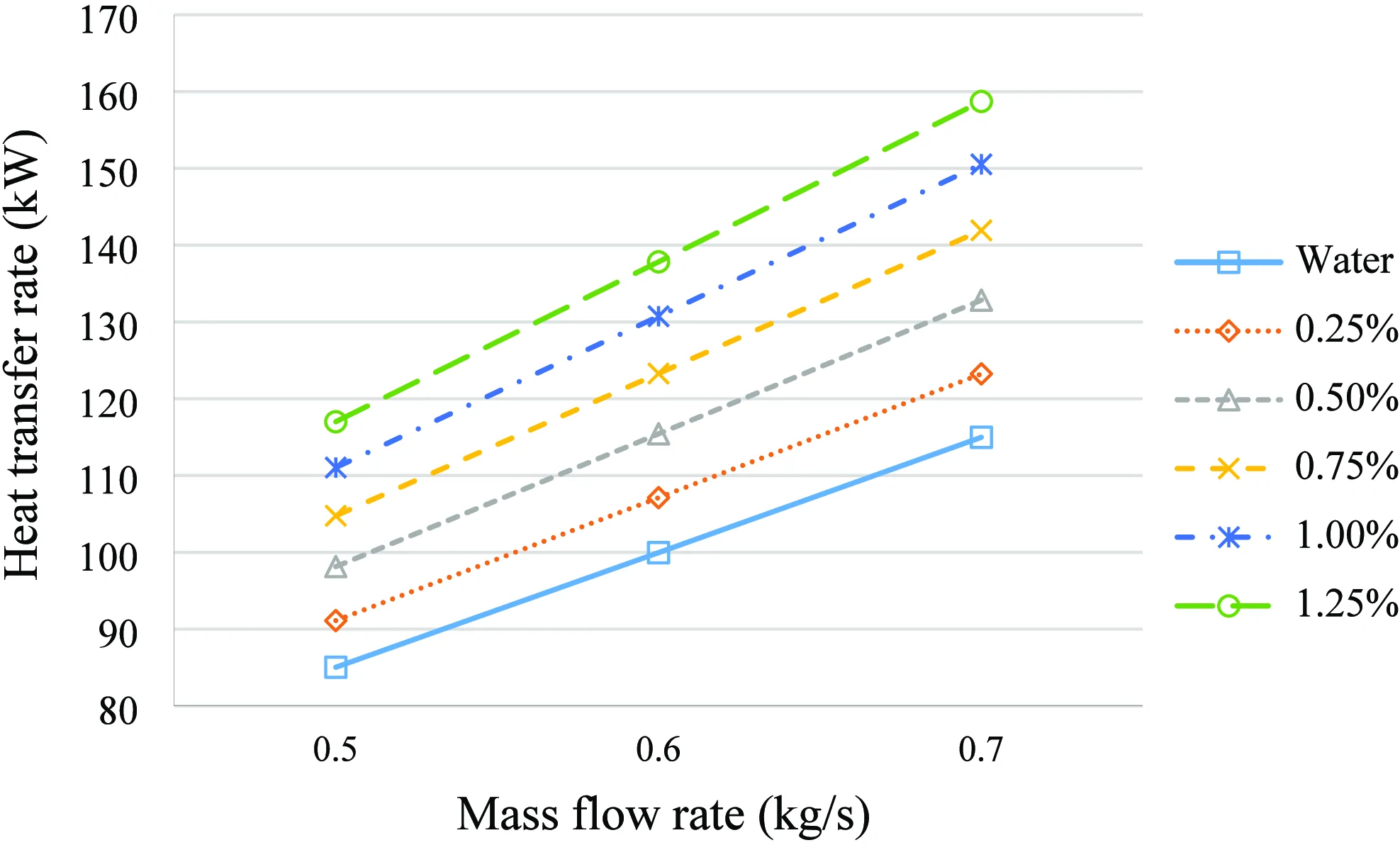
Heat transfer rate as a function of mass flow rate for different concentrations of Al_2_O_3_/water nanofluid.

Figure 4 shows that the higher the volume fraction of nanoparticles, the higher the heat-transfer rate tends to be. The most significant improvements are observed at a nanoparticle volume concentration of 1.25%, resulting in an average increase of 37.83% compared with pure water at the three different mass flow rates. For lower concentrations, the average increases in heat transfer are 7.16% at 0.25%, 15.47% at 0.50%, 23.32% at 0.75%, and 30.76% at 1.00%. These significant improvements are mainly associated with the synergistic influence of the improved thermal properties of the base fluid and the dispersed nanoparticles that form the nanofluid. However, the degree of improvement also depends on the type of nanoparticles used,^111^ their specific characteristics,^24^ their volumetric concentration, and the flow operating conditions.^112^

In addition, the analysis shows that the convective heat transfer rate of nanofluids rises as the mass flow rate increases. The percentage difference between the lowest and highest mass flow rates reaches approximately 35.64% for the nanofluid with a nanoparticle volume concentration of 1.25%. These results indicate that nanofluids exhibit a considerably higher efficiency in strongly turbulent flow regimes. This improvement is mainly attributed to the chaotic motion of water molecules and nanoparticles, which promotes better mixing between the two phases, thereby enhancing the transport of momentum, heat, and mass and ultimately improving heat transfer. This finding is consistent with the results reported by Ghazanfari et al.^48^ and Cruz et al.^68^

### 3.3 Heat Transfer Coefficient of Al_2_O_3_/Water Nanofluid

The influence of different nanofluid concentrations on the heat transfer coefficient is illustrated in Figure 5.

**Figure 5:**
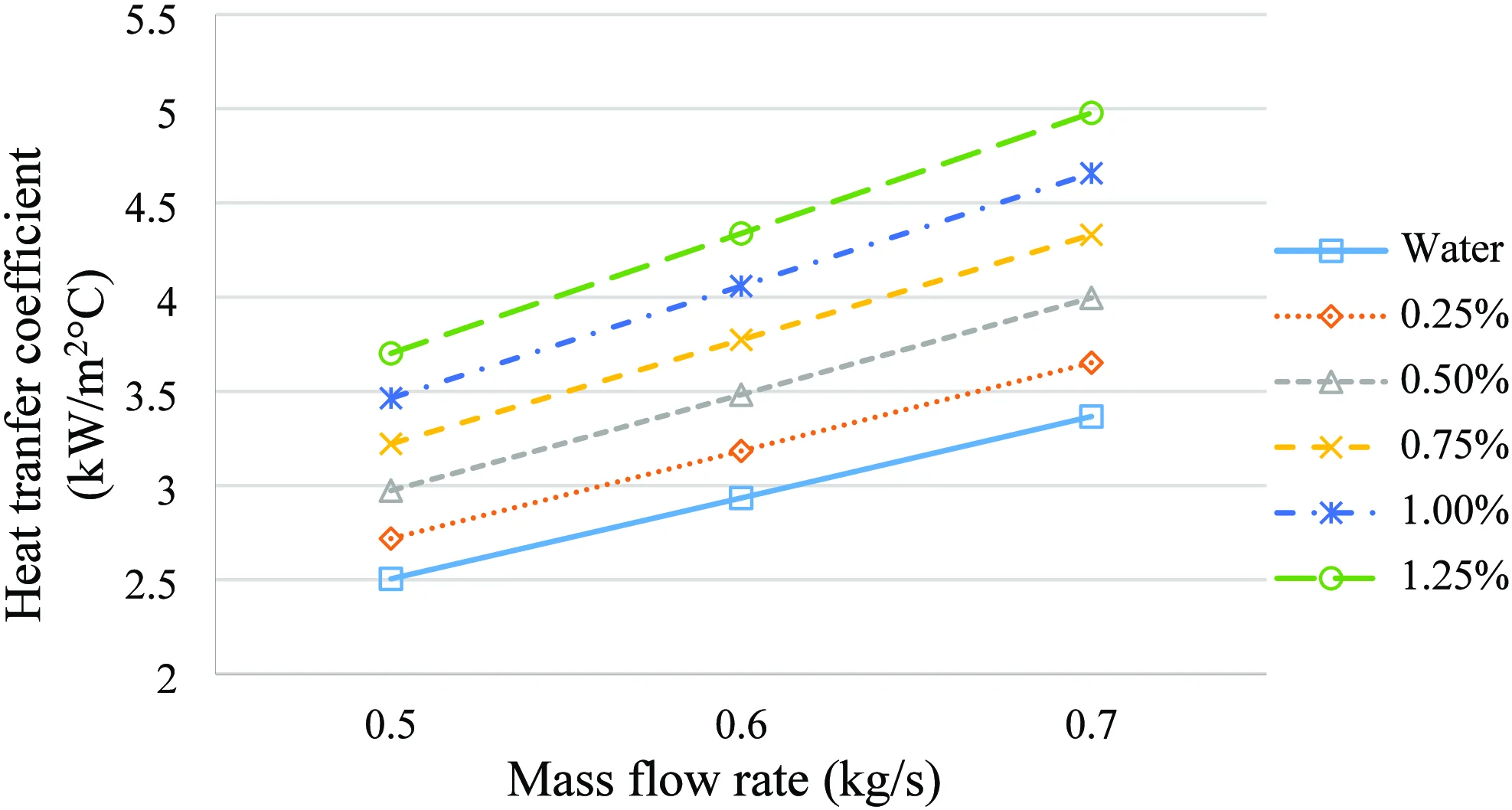
Heat transfer coefficient as a function of mass flow rate for different concentrations of Al_2_O_3_/water nanofluid.

Figure 5 shows that an increase in the mass flow rate leads to an improvement in the heat-transfer coefficient. This behavior is attributed to higher flow velocities, which reduce the thermal boundary layer thickness, thereby producing a sharper temperature gradient. Consequently, the reduced conductive resistance within this thinner layer increases the temperature difference between the tube wall and the bulk fluid, promoting a more homogeneous temperature profile near the heat-exchange interface and resulting in more efficient heat transfer.^110^

In addition, an increase in the heat-transfer coefficient is observed as the concentration of nanoparticles increases. This effect is primarily linked to the improvement in thermal conductivity since convective heat exchange depends on the capacity of the colder fluid to uptake heat when exposed to a hotter surface. The presence of nanoparticles improves the thermal conductivity of the coolant, thereby facilitating a more efficient heat transfer.^32^ However, the highest percentage increase occurs across all nanofluid mass flow rates when the nanoparticle volume concentration is 1.25%, as shown in Figure 5. Compared with water, this increase corresponds to an average of 47.84%. Additionally, for lower concentrations, the increase is 8.51% at 0.25%, 18.70% at 0.50%, 28.62% at 0.75%, and 38.32% at 1.00%.

### 3.4 Pressure Drop of Al_2_O_3_/Water Nanofluid

Figure 6 shows the influence of variations in the nanofluid concentration on the pressure drop.

**Figure 6:**
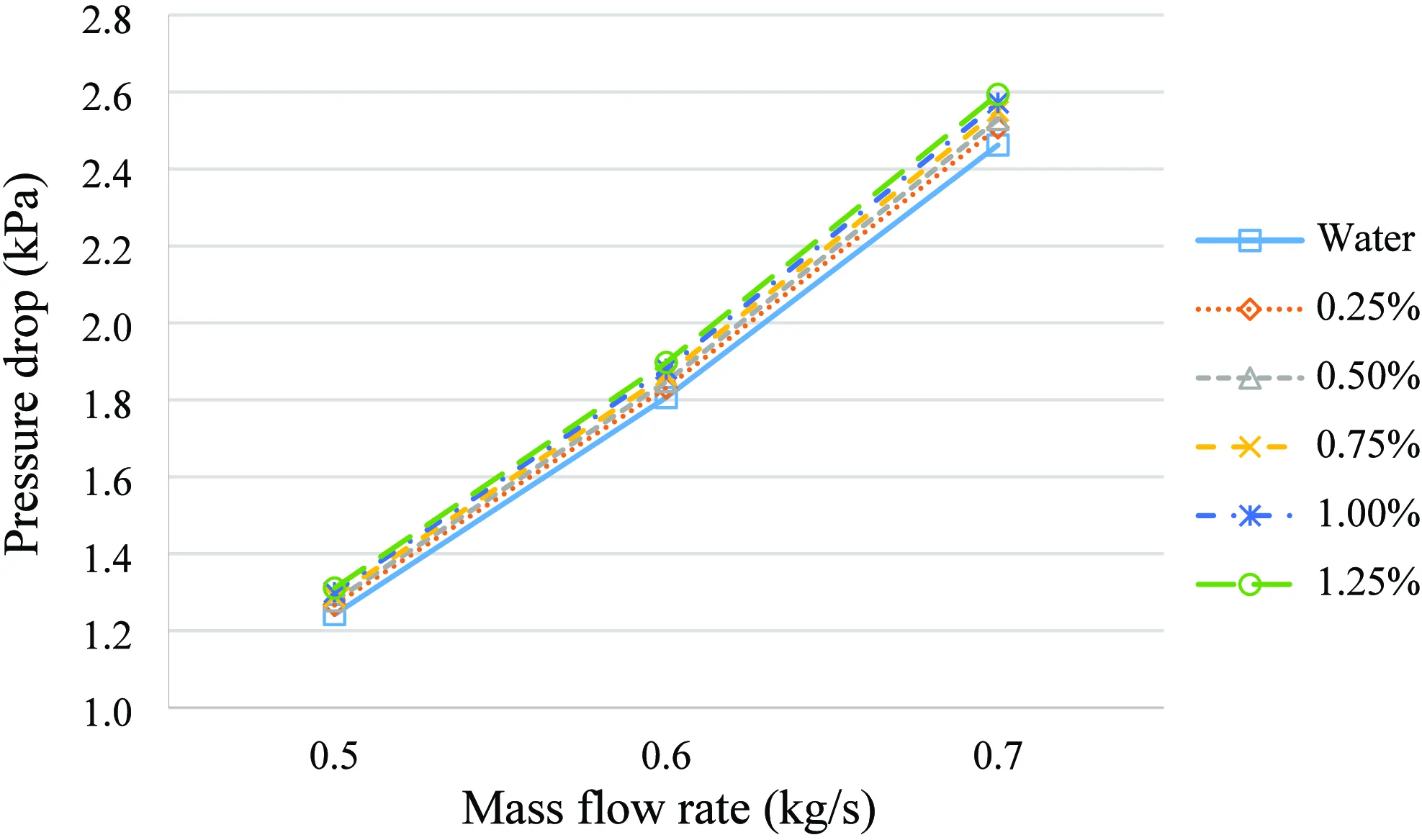
Pressure drops as a function of mass flow rate for different concentrations of Al_2_O_3_/water nanofluid.

In heat exchangers, pressure drop denotes the decrease in fluid pressure as it flows through the equipment. This reduction is caused by friction between the viscous fluid and the system walls, as well as internal friction losses between fluid layers. In turbulent flows — characterized by more intense and chaotic interactions between the fluid layers — higher resistances are generated, resulting in a greater pressure drop compared to laminar flows.^110^

Figure 6 also shows a slight increase in the pressure drop as the nanoparticle concentration increases. This pressure loss is attributed to the increase in viscosity since fluid viscosity is one of the primary causes of pressure drop, in addition to the increase in flow velocity. Because the nanoparticles significantly enhance this property, the effect becomes more pronounced.^111^ In addition, Figure 6 shows that the highest percentage pressure drop occurs at a nanoparticle volume concentration of 1.25%. Compared with pure water, this corresponds to an average increase of 5.30%. For lower concentrations, the pressure drop increases by 1.72% at 0.25%, 2.61% at 0.50%, 3.51% at 0.75%, and 4.41% at 1.00% volume concentrations.

Furthermore, although the increase in pressure drop becomes more noticeable at higher nanoparticle concentrations, the corresponding impact on pumping power remains relatively moderate within the investigated operating conditions. To further evaluate the practical feasibility of the proposed nanofluid configurations, the pumping power requirement was analyzed as a function of both mass flow rate and nanoparticle concentration.

Figure 7 illustrates the variation of the pumping power for the investigated cases. As expected, the pumping power increased with an increasing mass flow rate due to the higher pressure losses associated with greater flow velocities. Likewise, a slight increase in pumping power was observed as the nanoparticle concentration increased because of the corresponding increase in effective viscosity.

**Figure 7:**
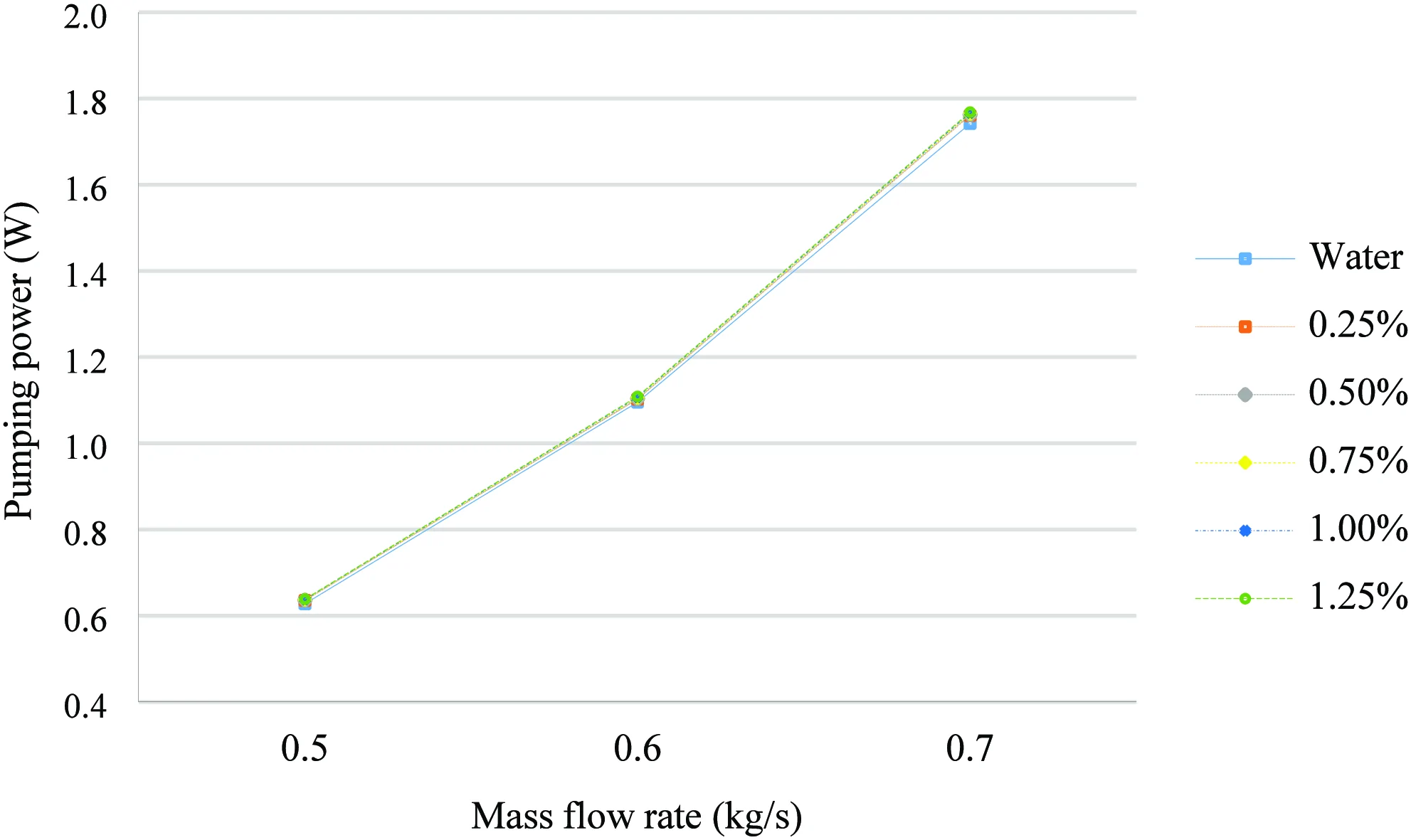
Pumping power of Al_2_O_3_/water nanofluid at different volume concentrations and mass flow rate.

However, the obtained hydraulic penalty remained comparatively small throughout the investigated concentration ranges. For instance, at a mass flow rate of 0.7 kg/s, the pumping power increased from 1,742W for water to 1,768W for the 1.25% nanofluid, corresponding to an increase of approximately 1.5%. This relatively small increase indicates that dilute Al_2_O_3_/water nanofluids can improve the heat transfer performance without imposing excessive additional energy consumption. From an industrial perspective, these results indicate a favorable thermohydraulic trade-off, since the thermal enhancement achieved with nanofluids was accompanied by only a limited increase in pumping power requirements. It should be noted that the increase in pumping power may be either minor or significant, depending on the nanoparticle concentration, mass flow rate, heat exchanger configuration, and type, as reported in previous studies.^108,109,113,114^

Compared with recent studies employing passive geometric modifications, the present results indicate a favorable thermohydraulic compromise. At a nanoparticle volume concentration of 1.25%, the Al_2_O_3_/water nanofluid increased the heat transfer rate and heat transfer coefficient by 37.83% and 47.84%, respectively, while the pressure drop increased by only 5.30% and the pumping power by approximately 1.5%. Although direct quantitative comparisons should be interpreted cautiously because of differences in heat exchanger geometry, operating conditions, and performance indicators, the present trends are consistent with recent investigations of twisted, corrugated, conical, and multihelical configurations, which also reported improved thermal performance accompanied by frictional and hydraulic penalties.^38–41^

From an industrial perspective, the observed increase in the pressure drop remained relatively modest, even at the highest nanoparticle concentration investigated. The corresponding increase in pumping power was limited, suggesting that the thermal enhancement achieved by the nanofluid may be greater than the additional hydraulic requirements. Therefore, the proposed low-concentration Al_2_O_3_/water nanofluids could represent a feasible alternative for shell-and-tube heat exchangers, where improved thermal performance is desired without substantial increases in operating costs. Similar observations were reported by Singh, Verma, and Ghosh,^115^ who investigated the thermal performance of an industrial heat exchanger using graphene-based nanofluids at concentrations of 0.1%, 0.25%, 0.5%, 0.75%, and 1.0%. Their results showed that increasing the nanoparticle concentration led to higher pressure drops and, consequently, greater pumping requirements. However, the authors concluded that the resulting thermal enhancement was more significant than the associated hydraulic penalty and that this effect could be compensated for by operating the pump at a higher head. Nevertheless, they emphasized that the pressure drop remains an important design parameter, as it directly affects pumping requirements, piping design, energy consumption, and overall system cost. Therefore, further studies under different operating conditions and nanofluid formulations are required to better assess the industrial applicability of nanofluid-based heat-transfer systems.^116^

It is important to note that the present thermohydraulic assessment is based on first-law performance indicators, namely, heat transfer rate, heat transfer coefficient, pressure drop, and pumping power. Although these quantities provide useful design-oriented information, they do not directly quantify the irreversibilities associated with heat transfer across finite-temperature differences and viscous dissipation. Entropy generation analysis has been increasingly recognized as an effective second-law-based method for evaluating the compromise between thermal enhancement and frictional losses in heat exchangers using advanced working fluids and passive enhancement techniques. Therefore, future studies should incorporate local and global entropy generation rates to identify the optimum nanoparticle concentration and operating conditions from a second law perspective. This approach has been emphasized in recent studies on enhanced heat-transfer systems and nanofluid-based thermal devices.^117,118^

### 3.5 Analysis of Velocity, Pressure, and Temperature Fields Inside the Heat Exchanger Shell

The analysis of the velocity, pressure, and temperature contours extracted from the simulations carried out in the ANSYS Fluent software is based on sound physical principles. The velocity fields provide crucial information about the flow distribution and the formation of recirculations, while the pressure fields indicate variations in flow resistance along the heat exchanger. Temperature fields, on the other hand, offer detailed insights into the thermal gradients and heat transfer patterns within the system. This detailed visualization, supported by the scientific rigor of numerical simulation, enables an accurate assessment of the heat exchanger’s performance and informs the design of strategies for enhancing its thermal efficiency. Therefore, the objective of this section is to understand the flow behavior and thermal dynamics of the system, with the aim of gaining insights into how nanofluids influence the thermal performance of the heat exchanger.

#### 3.5.1 Velocity Field Analysis

The fluid flow behavior along the heat exchanger is illustrated through contours in Figure 8(a), corresponding to pure water, and Figure 8(b), corresponding to the nanofluid with a 1.25% volume concentration.

**Figure 8:**
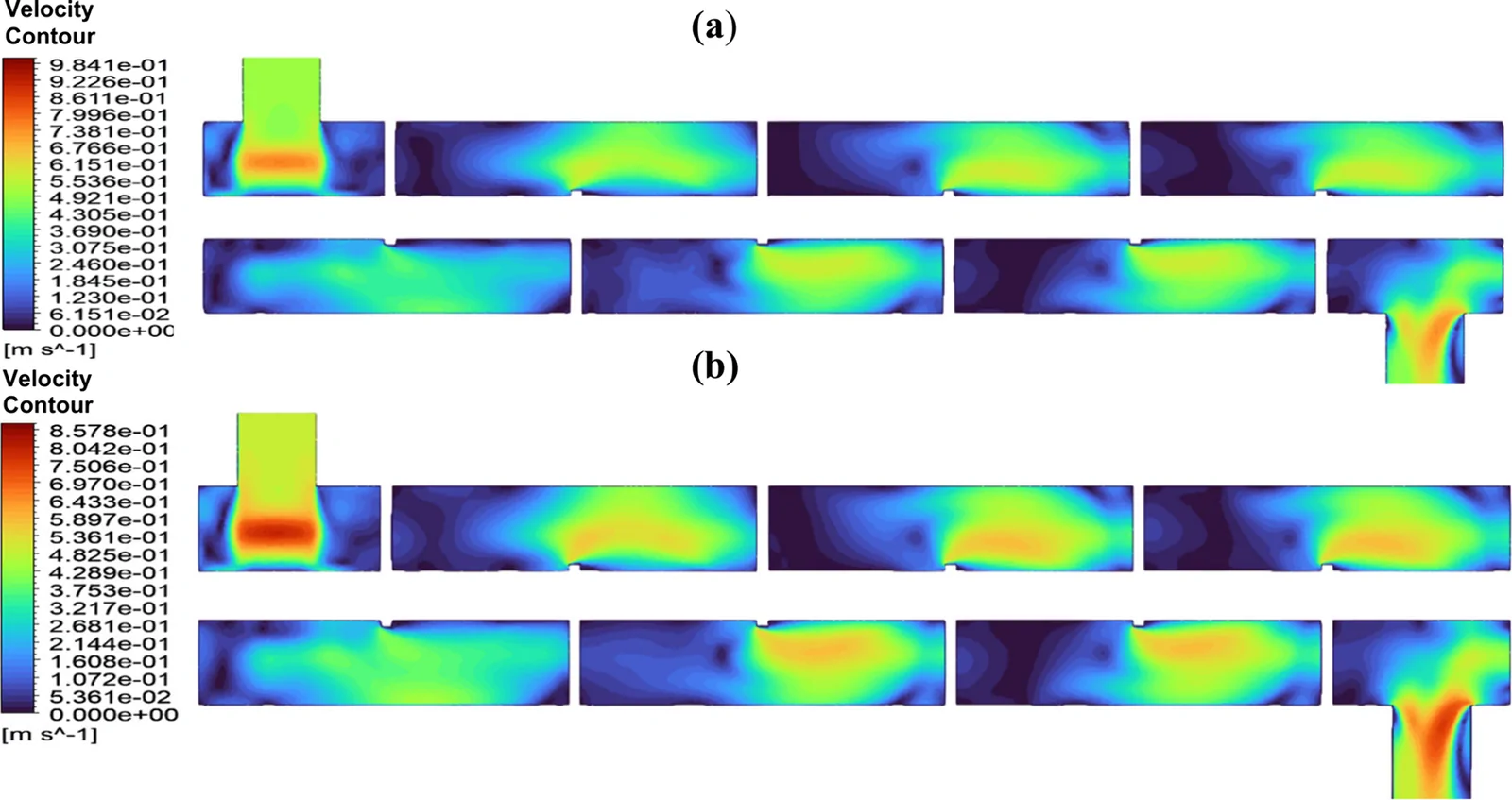
Velocity contour along the heat exchanger when operating with (a) pure water and (b) nanofluid (1.25% vol.) at a mass flow rate of 0.5 kg/s.

It can be observed that the velocities at the shell inlet and outlet are higher because of the smaller cross-sectional area in these regions. Additionally, a reduction in flow velocity is observed near the baffles, as a no-slip condition is applied to the baffle walls. Furthermore, the addition of solid Al_2_O_3_ nanoparticles to the base fluid results in a decrease in the flow velocity, as indicated by the velocity scales. This phenomenon is attributed to the increase in shear stress between the nanoparticles and the water molecules, which leads to an increase in the viscosity of the resulting nanofluid and, consequently, a reduction in its flow velocity.^110^

Figure 9 shows similar streamlines between water and the nanofluids. This flow behavior is expected since the nanofluid was modeled as a homogeneous fluid.^119^ In this approach, the dispersed phase does not significantly alter the transport characteristics of the continuous phase.

**Figure 9:**
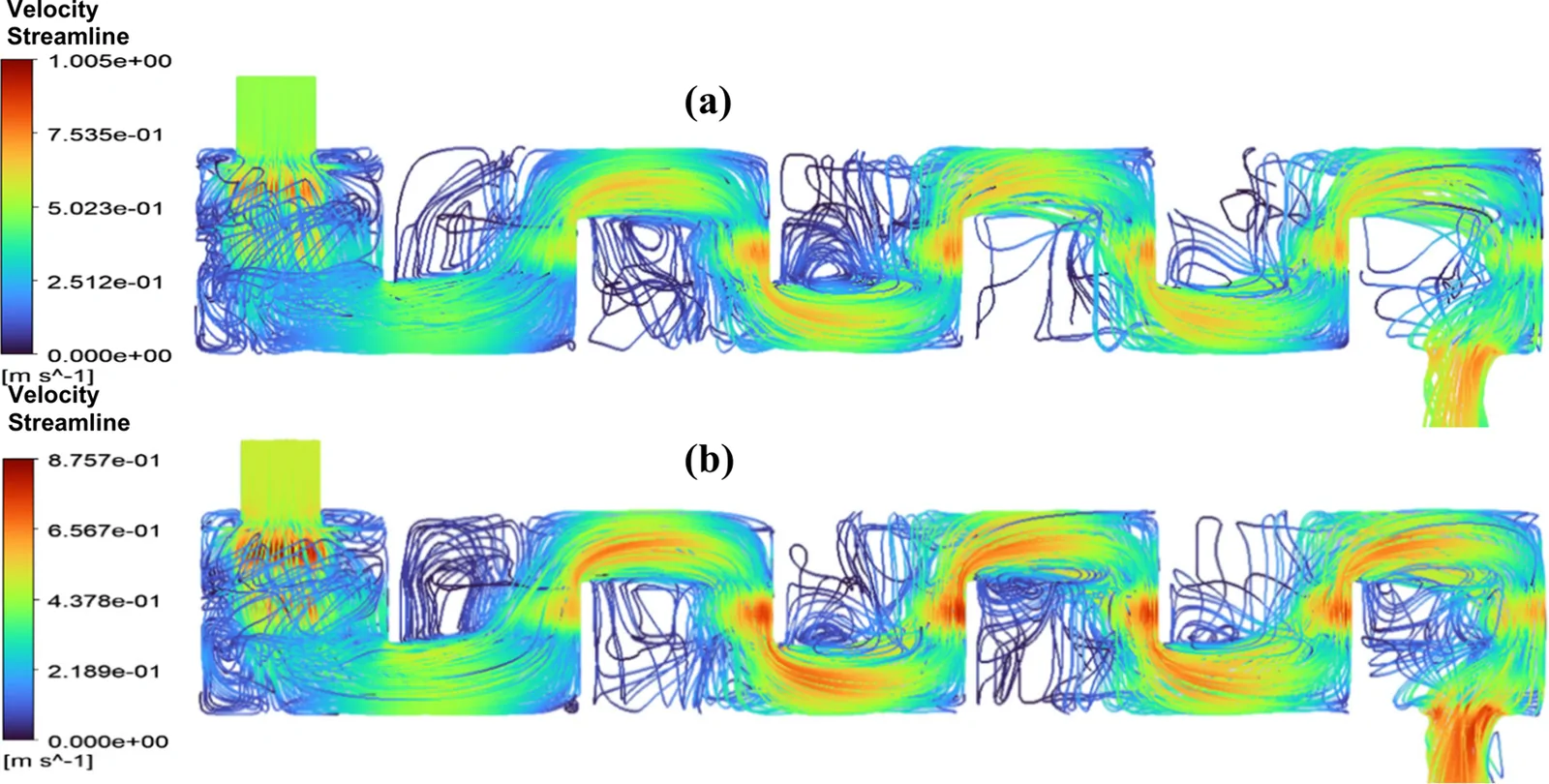
Streamlines for the heat exchanger operating with (a) pure water and (b) nanofluid (1.25%) at a mass flow rate of 0.5 kg/s.

The recirculation zones can be identified through the streamlines, which contribute to an increase in the heat-transfer coefficient, although they are associated with notable pressure losses.^120^ These recirculation zones are formed due to the impact of the flow against the baffle walls and tend to be more pronounced at higher mass flow rates, enhancing the parameters associated with heat transfer.^121^ Furthermore, an increase in the mass flow rate enhances fluid momentum, which intensifies shear stresses and turbulence, thereby leading to a higher dissipation of mechanical energy as a pressure drop.

Physically, the flow is redirected to bypass the baffle surface (spreading upward, downward, or around it), where its velocity decreases near the stagnation zone, converting the kinetic energy into static pressure. This mechanism generates an adverse pressure gradient that induces boundary-layer separation along the solid surface of the baffle, resulting in the formation of recirculation zones on the downstream side of the six baffles.

Comparable recirculation zones appear in the streamlines of the other cases investigated. In practice, this fluid stagnation behind the baffles not only results in pressure drop penalties but also can induce corrosion on the tube walls. Consequently, mitigating the occurrence of these zones is critical. Furthermore, the formation of recirculation zones is strongly influenced by the heat exchanger configuration, particularly the baffle spacing and baffle cut.^12^

Figure 10 presents the velocity profiles at different cross-sectional planes throughout the heat exchanger when operating with an Al_2_O_3_/water nanofluid at a 1.25% volumetric concentration. Because of the shell-side arrangement and the inclusion of baffles, the velocity profile across these sections of the exchanger does not reach a fully developed state. Interestingly, the profiles for both water and nanofluid in all cases are similar, with the fluid near the outer tubes exhibiting a higher velocity gradient compared to that near the center of the shell. In addition, velocity vector plots (Figure 11) are provided to better illustrate the influence of the nozzle and tube configuration on the pressure and temperature fields.

**Figure 10:**
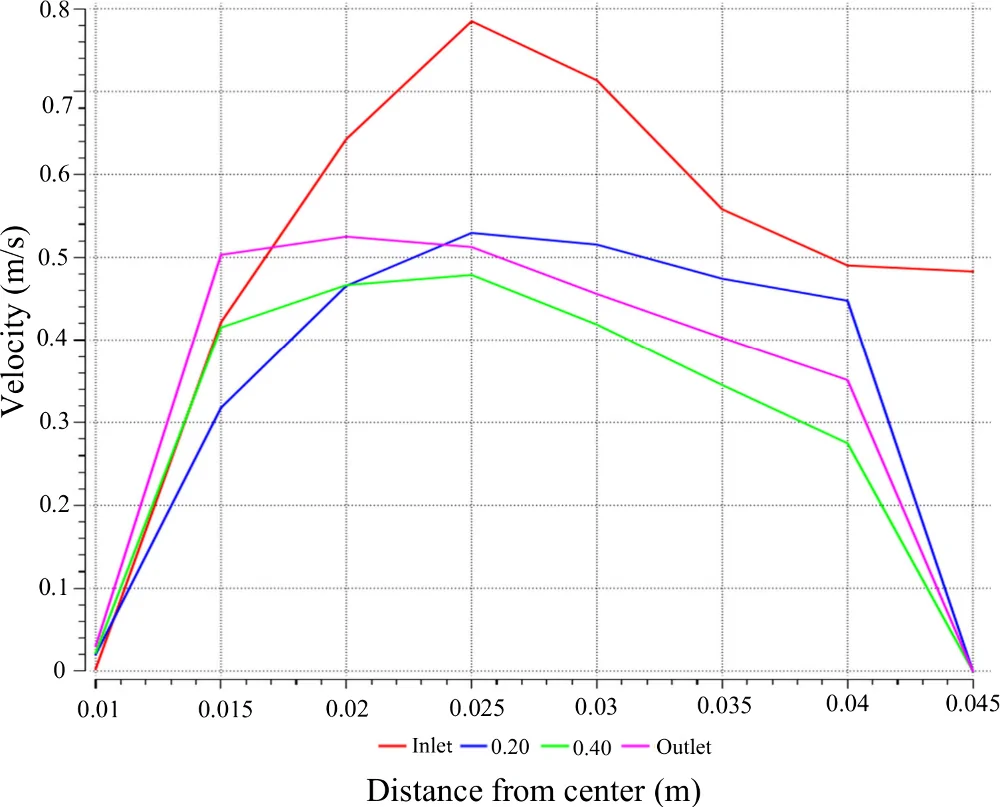
Cross-sectional profile for Al_2_O_3_/water nanofluid (1.25% vol.) at a mass flow rate of 0.5 kg/s.

**Figure 11:**
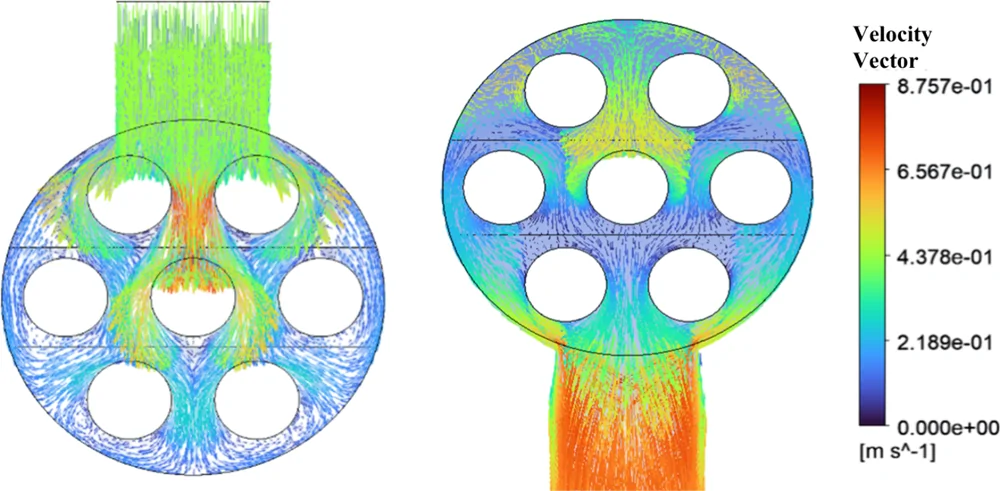
Cross-sectional profile for Al_2_O_3_/water nanofluid (1.25%) vol. at a mass flow rate of 0.5 kg/s.

#### 3.5.2 Pressure Field Analysis

Figure 12 shows the pressure drop as a function of the heat exchanger length for the five nanoparticle volume concentrations tested at the lowest mass flow rate (0.5 kg/s). In all cases, significant pressure gradients can be observed along the heat exchanger, which are likely due to the presence of baffles inside the shell of the specific tube arrangement. Figure 11 also shows that the pressure drop presents a negligible dependence on the Al_2_O_3_ nanoparticle concentration in the water along the heat exchanger length. Figure 13 shows the pressure contours along the heat exchanger for the case with 1.25% vol. of Al_2_O_3_/water nanofluid.

**Figure 12:**
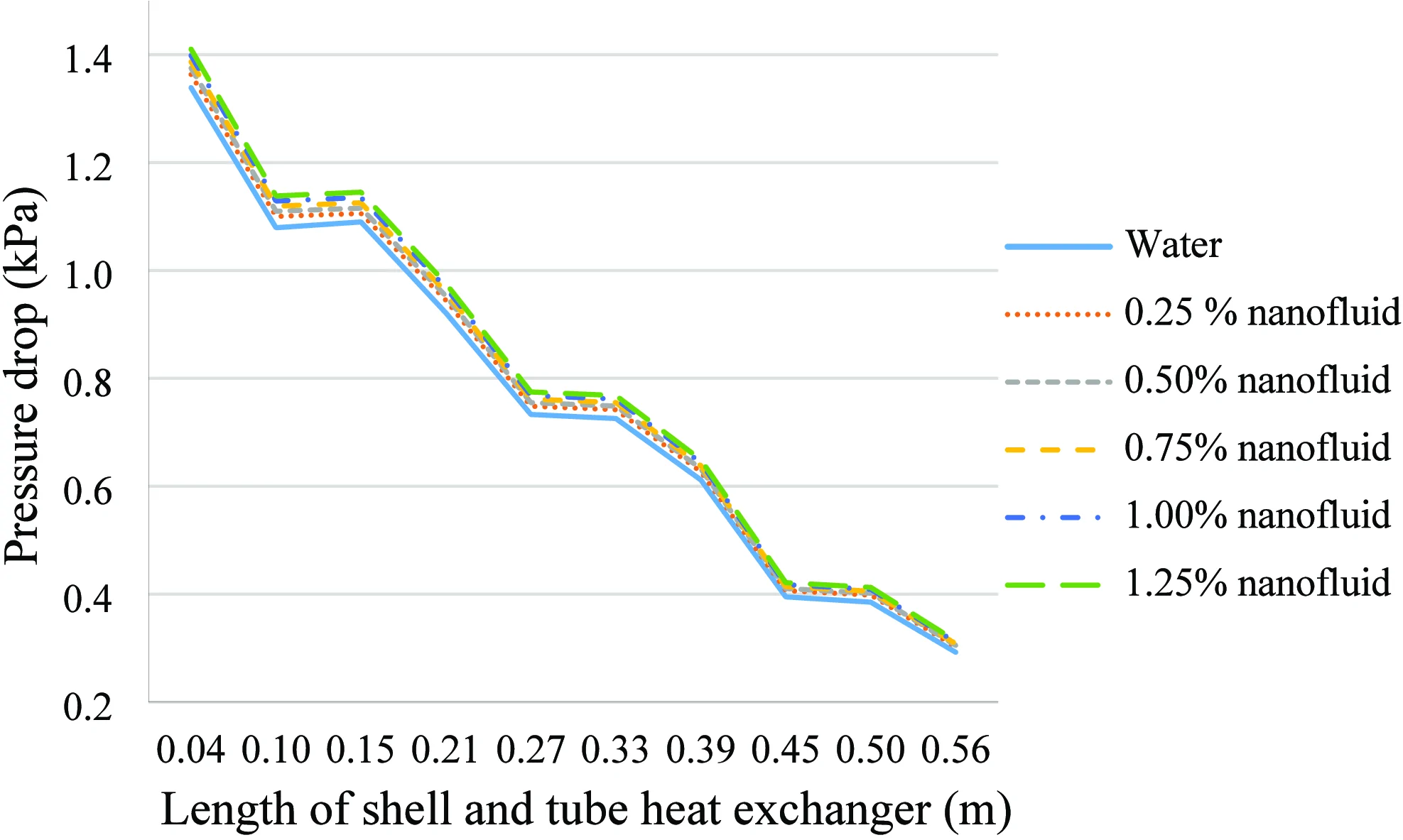
Pressure profile along the heat exchanger for the Al_2_O_3_/water nanofluid (1.25%) with a mass flow rate of 0.5 kg/s.

**Figure 13:**
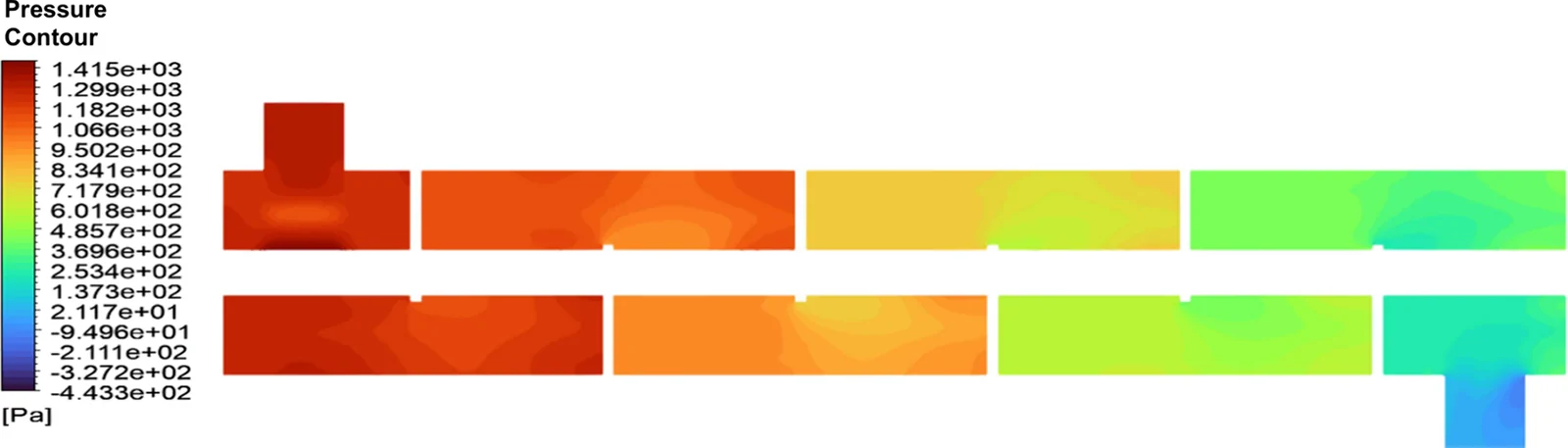
Pressure contour along the heat exchanger for Al_2_O_3_/water nanofluid (1.25%) at a mass flow rate of 0.5 kg/s.

As shown in Figure 13, the highest pressure drop occurs at the inlet of the heat exchanger, which can be attributed to the geometric features and flow development at the system entry. Upon entering, the fluid encounters resistances that hinder its progression through the equipment, primarily driven by flow-direction changes, recirculations, and wall friction. The latter is slightly augmented with increasing Al_2_O_3_ nanoparticle concentrations due to the associated rise in fluid viscosity.^122^ The high velocity at which the flow enters makes it possible to overcome these resistances, making it possible for it to flow naturally through the equipment, however, with a higher penalty in pressure drop, as illustrated in Figure 13.

In the regions where recirculations occur (see Figure 8), the flow becomes more turbulent, and high local pressures are observed due to fluid impingement against the solid surfaces of the device, leading to the dissipation of kinetic energy. Consequently, this continuous degradation of mechanical energy results in a progressive reduction in static pressure along the equipment, as shown in Figure 13.

#### 3.5.3 Temperature Field Analysis

To analyze the thermal distribution, contour plots of temperature were obtained along the heat exchanger length and on selected cross-sectional planes, highlighting the steady-state behavior of both water and the nanofluid. Figure 14 shows the differences between the cross-sectional temperature contours of pure water and the nanofluid with 1.25% vol. Al_2_O_3_. The selected positions show the thermal behavior of the fluid in the inlet and outlet regions and also on the surfaces before, after, and between the baffles. As expected, the fluid temperature is uniform in the inlet region. Figure 14 shows that the temperature differences in the cross sections between pure water and the nanofluid are pronounced. Starting from 0.20 m, a temperature gradient spanning from 45 to 73 °C develops near the tube walls for the nanofluid. At 0.40 m, this discrepancy becomes even higher, such that the lower cross-sectional region of the fluid layer exhibits a higher temperature range (from 73 to 85 °C) for the nanofluid compared to the thermal contour of water at the same axial position.

**Figure 14:**
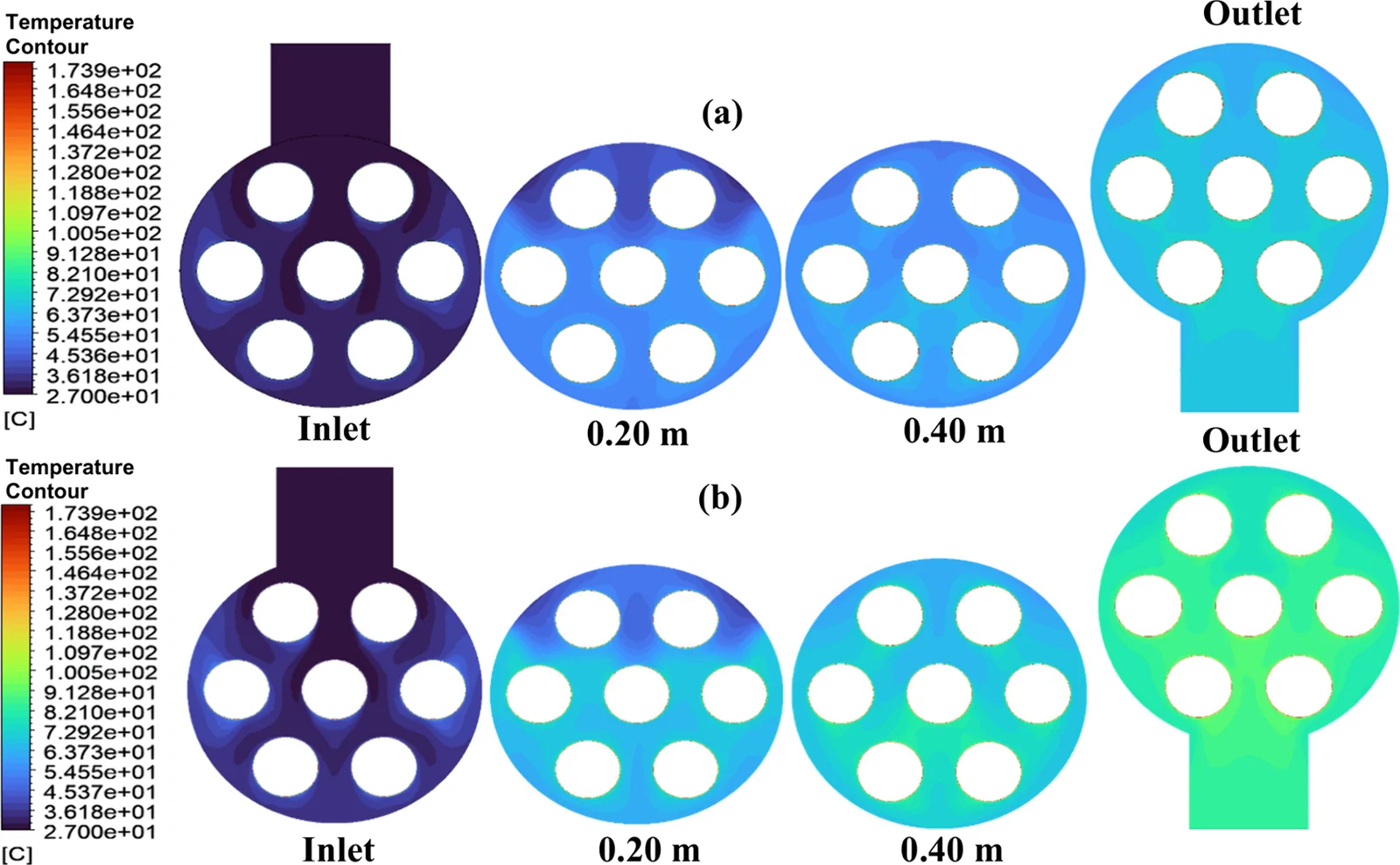
Cross-sectional temperature contours for (a) pure water and (b) 1.25% vol. Al_2_O_3_/water nanofluid.

According to Wang et al.,^123^ this behavior can be explained by the concept of specific heat, also referred to as specific heat capacity, which is a physical property that represents the amount of heat required to raise the temperature of a unit of mass of a substance by one degree. In this context, it is expected that the use of nanofluids to enhance heat transfer performance leads to a reduction in specific heat. This occurs because the high thermal conductivity introduced by the addition of solid nanoparticles to the base fluid enables a more efficient heat conduction. Consequently, the specific heat capacity of the nanofluid is reduced, which implies that a lower amount of energy is required to increase its temperature.^124^ This behavior is consistent with that reported by Cruz et al.,^68^ who studied the application of nanofluids in shell-and-tube heat exchangers through CFD analysis.

Figures 15 and 16 show the general cross-sectional temperature profiles. The curves illustrate the thermal behavior of the fluids with respect to the shell radius, which, regardless of the type of fluid used, becomes nearly uniform from approximately 0.015 m outward. This behavior is attributed to the fact that the flow within the shell does not move in a perfectly stratified manner but rather exhibits turbulent characteristics. Such turbulence promotes improved fluid mixing and, consequently, a more homogeneous temperature distribution in the radial direction. Although there are no immediate differences in the shape of the temperature profiles between water and nanofluid, the overall temperature of the nanofluid is consistently higher than that of water, with this increase becoming more pronounced as the concentration of nanoparticles rises.

**Figure 15:**
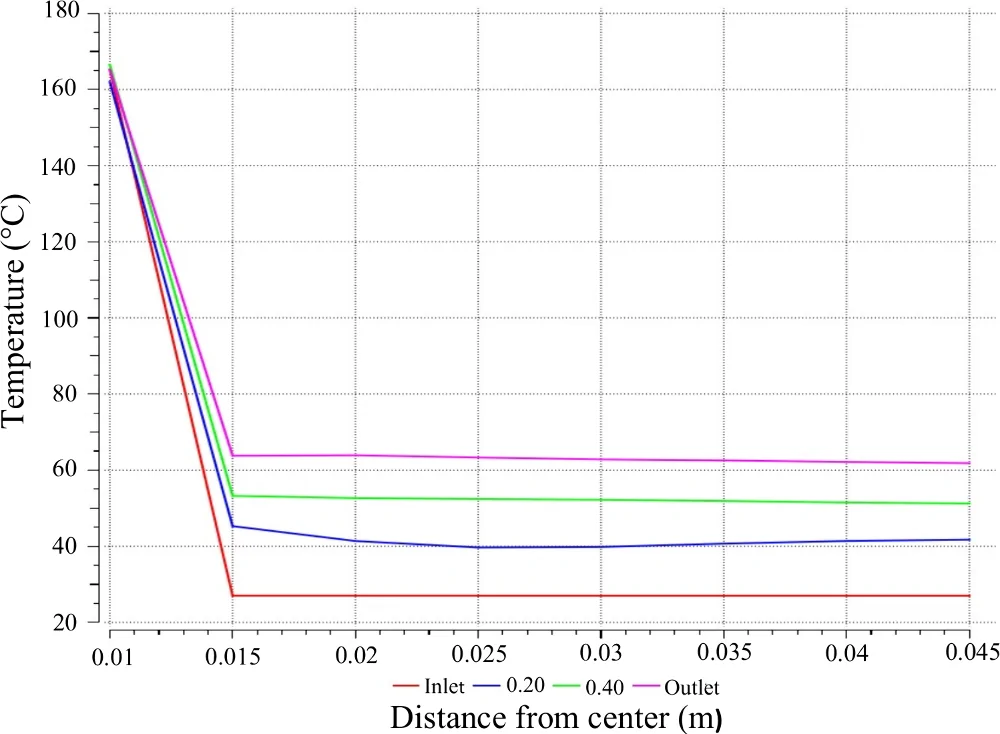
Cross-sectional temperature profile for pure water at a mass flow rate of 0.5 kg/s.

**Figure 16:**
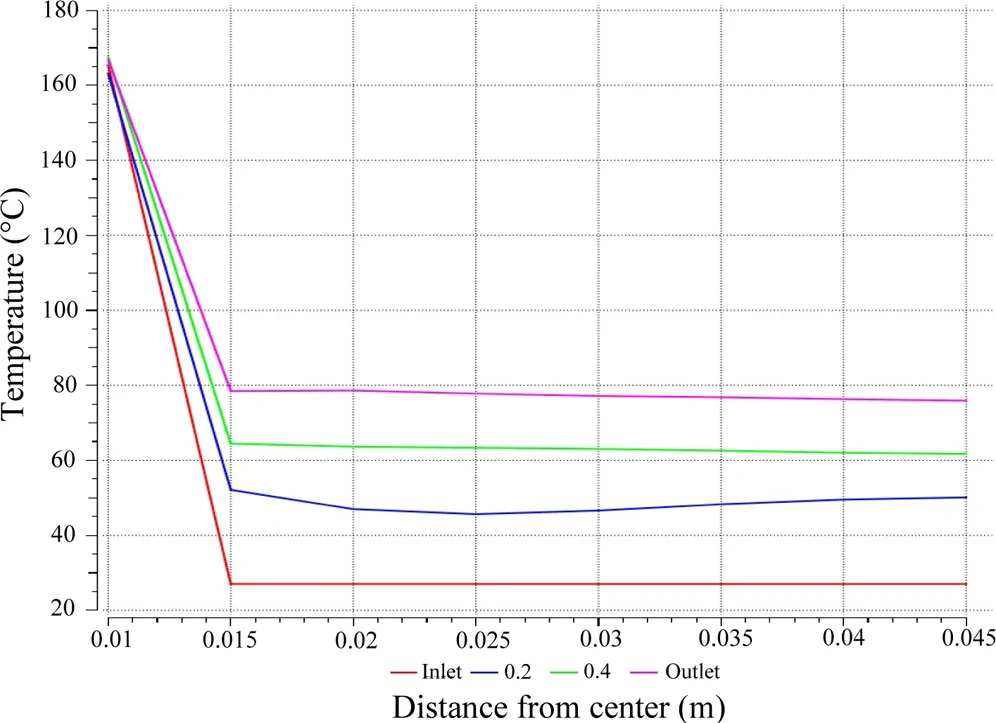
Cross-sectional temperature profile for nanofluid (1.25%vol) at a mass flow rate of 0.5 kg/s.

Similar to the findings reported by Cruz et al.,^68^ in all analyzed cases, it is evident that the fluid in the vicinity of the tubes located in the central region of the heat exchanger experiences a greater temperature increase compared to the fluid near the outer tubes. This behavior can be attributed to the recirculation zones that formed in this area. This behavior can be attributed to the recirculation zones that form in this region. The lower flow velocities lead to longer fluid residence times in contact with the outer surfaces of the tubes, which are maintained at elevated temperatures, thereby enhancing the heat transfer.

Figure 17 presents the temperature contour along the symmetry plane of the heat exchanger for the case of 1.25% vol Al_2_O_3_/water nanofluid. As previously mentioned, at approximately 0.40 m from the inlet, the lower region exhibits a higher temperature range compared to the surrounding zones outside the main flow path. Figure 18 shows the thermal behavior of the fluid through the average temperature profile at a mass flow rate of 0.5 kg/s for five nanoparticle volume concentrations.

**Figure 17:**
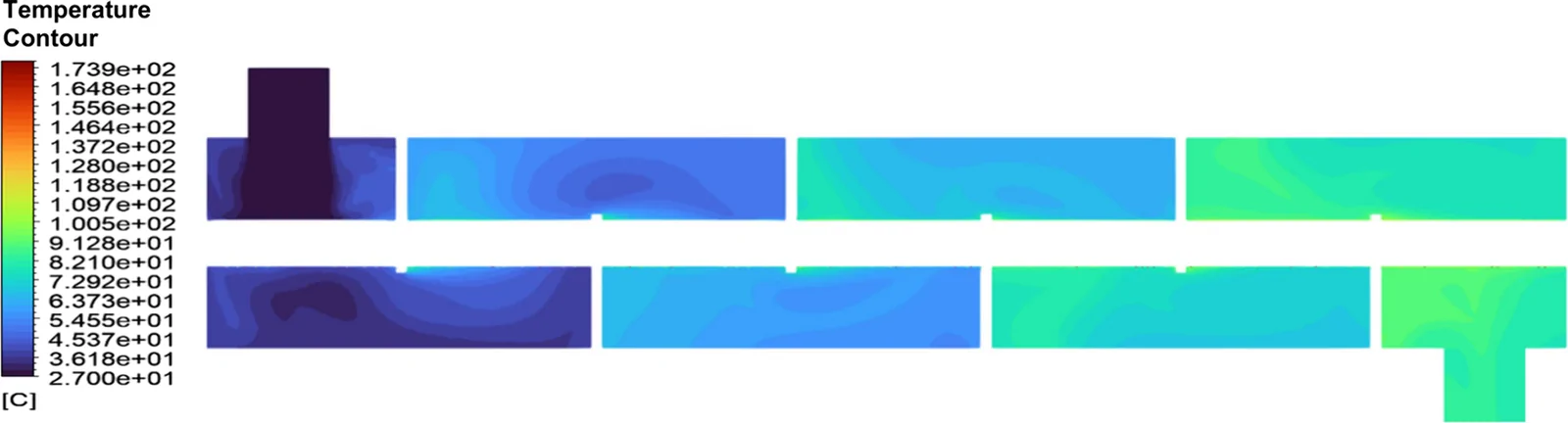
Temperature contour throughout the heat exchanger for nanofluid Al_2_O_3_/water (1.25%vol) at a mass flow rate of 0.5 kg/s.

**Figure 18:**
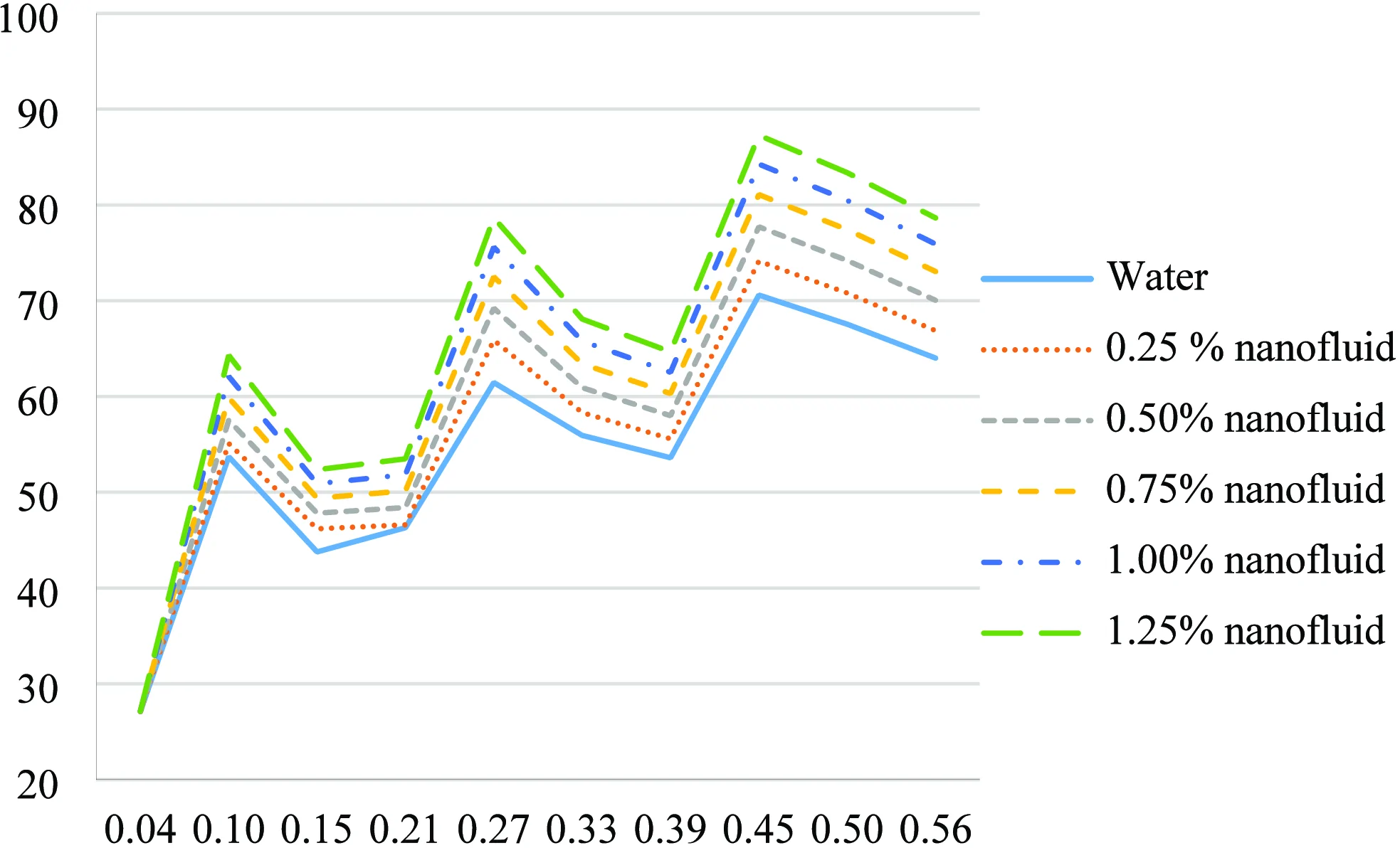
Average temperature profiles as a function of the shell and tube heat exchanger length for different concentrations of Al_2_O_3_/water nanofluid.

The nanofluid at 1.25% vol. presented the steepest temperature gradient, a factor that justifies the higher heat-transfer rate obtained under these conditions. It is worth noting that for the remaining cases, the behavior of the average temperature distribution along the heat exchanger length was similar, albeit with lower temperature values. These results are consistent with those reported by Ozden and Tari,^12^ who indicated that the shell-side flow dynamics significantly influence the development of the fluid temperature profile.

For a mass flow rate of 0.5 kg/s, the thermal profile of the 1.25% vol nanofluid exhibited a higher temperature gradient and, consequently, a higher outlet temperature compared to its water counterpart. This demonstrates the improvement in the heat transfer performance resulting from the use of the nanofluid. For all cases, heat transfer occurred predominantly in the region between the inlet and approximately 0.10 m along the heat exchanger length, as indicated by the steep temperature gradients shown in Figure 18. The fluid temperature continuously increases up to around 80% of the heat exchanger length, beyond which the temperature gradient gradually diminishes toward the outlet nozzle.

### 3.6 Regression Model

In this section, a multiple linear regression model is proposed to estimate the shell-side heat transfer rate (dependent variable) as a function of the mass flow rate and volumetric concentration of nanoparticles (independent variables) used. The regression equation serves as the mathematical tool that models the relationship between these variables. In the proposed model, dimensionless values for mass flow rate (*m*), volume fraction (*C*), and heat transfer rate (*Q*) are determined by applying eq 27.^125^

27 $$Q=\mathrm{Q̇}/Q_{3},,C=Ċ/C_{3},,m=ṁ/m_{3}$$

where *Q̇*, *Ċ*, and *ṁ* refer to the normalized heat transfer rate on the shell side of the heat exchanger, the normalized volume fraction of the nanofluid, and the mass flow rate, respectively. *Q*_3_, *C*_3_, and *m*_3_ refer to the minimum heat transfer rate on the shell side of the heat exchanger, the minimum volume fraction of the nanofluid (0.25%), and the minimum mass flow rate (0.5 kg/s), respectively. The regression structure was adapted from the correlations proposed by Hu et al.^125^ and Nazari et al.^126^ as follows:

28 $$y=a_{0}+b_{1}x_{1}+b_{2}x_{2}+c_{1}x_{1}x_{2}+f_{1}\exp^{d_{1}x_{1}}+f_{2}\exp^{d_{2}x_{2}}+\in$$

29 $$y=22.892+156.189x_{1}+9.824x_{2}+47.611x_{1}x_{2}+10.911\exp^{-1.5x_{1}}-21.358\exp^{0.3x_{2}}$$

where *y* represents the heat transfer rate on shell side, *a*_0_ denotes the intercept or constant term of the model, *c*_1_ is the coefficient for the interaction term, *x*_1_ and *x*_2_, representing the combined influence of both variables, *x*_1_ is the mass flow rate, and *x*_2_ is the nanoparticle volume concentration. The coefficients *b*_1_ and *b*_2_ correspond to the linear effects of *x*_1_ and *x*_2_, respectively. The terms *f*_1_ and *f*_2_ are the coefficients associated with the exponential components, while *d*_1_ and *d*_2_ are the exponent parameters that define the rate of variation of the exponential terms for *x*_1_ and *x*_2_, respectively. Finally, ϵ represents the model’s error term.

To evaluate the predictive capability and robustness of the regression model (eq 29), a validation subset was utilized. The multiple linear regression was trained using a data set of 12 cases obtained from the ANSYS simulations, while the remaining 6 independent cases were reserved exclusively for model testing.

Response surface methodology (RSM) was employed to develop a regression model describing the behavior of the system. Figure 19 shows the surface obtained for the above correlation. The response surface generated from the regression model was used both to predict the heat transfer rate under different operating conditions and to visualize the optimal combination of parameters identified by the regression equation, which corresponded to a mass flow rate of 0.7 kg/s, a nanoparticle volume concentration of 1.25%, and a heat transfer rate of 158.70%. The R-squared of the proposed regression is 0.9998, and the residual standard error is 0.32, indicating the reliability of the model.

**Figure 19:**
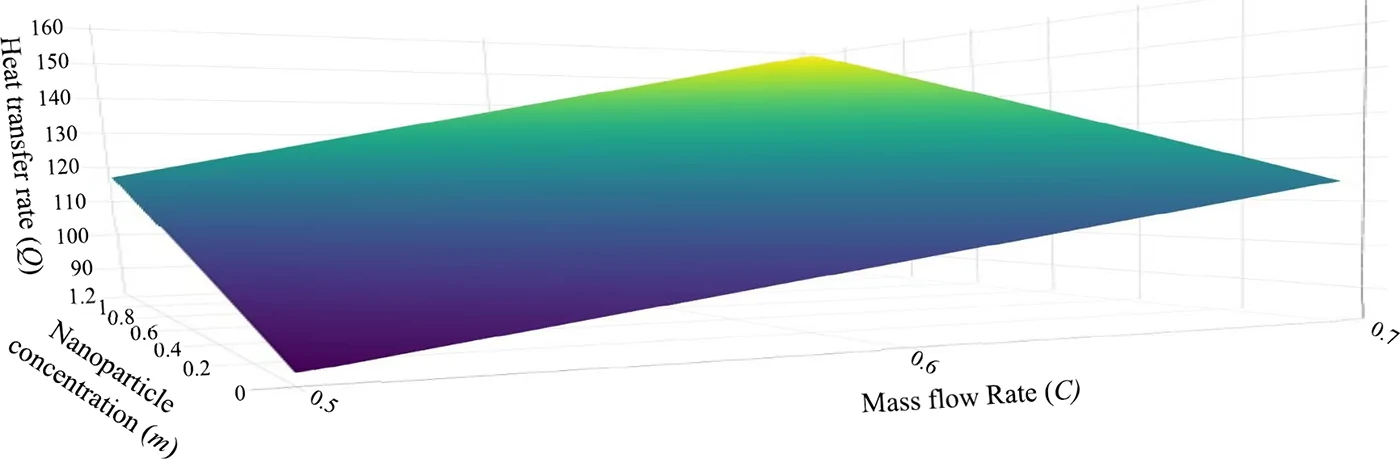
Predicted results of the regression equation obtained from RStudio Software.

The proposed correlation was developed specifically for the operating conditions investigated in the present study and demonstrated excellent predictive capability with an R^2^ value of 0.9998 and a residual standard error of 0.32. Its mathematical structure was adapted from regression approaches previously reported in the literature for predicting the thermal performance of nanofluid-based heat transfer systems. Nevertheless, a direct quantitative comparison among the correlations is limited by differences in heat exchanger geometry, working fluid, nanoparticle concentration range, flow conditions, and selected dependent variables. Within the investigated ranges, the present correlation provides a reliable predictive tool for estimating the shell-side heat-transfer rate of the Al_2_O_3_/water nanofluid system.

It should be emphasized that the proposed regression model is valid only within the investigated ranges of the mass flow rate and nanoparticle volume concentration. Extrapolation beyond these limits may lead to unreliable predictions and would require additional CFD or experimental validation.

## 4 Conclusions

This work evaluated the impact of using Al_2_O_3_/water nanofluids on enhancing the heat-transfer rate in a shell-and-tube heat exchanger. It can be concluded that correlation-based approaches show an adequate agreement with the CFD results. While these correlations may suggest potential design deficiencies, CFD simulations enable the identification of their origins and locations. Furthermore, the Kern method systematically underestimates the heat transfer coefficient values, resulting in a larger percentage deviation compared with the CFD results. For this reason, the baseline comparisons (using water alone) were carried out using the Bell-Delaware method, which incorporates correction factors and therefore provides greater accuracy.

CFD analyses demonstrated that the addition of nanoparticles to the base fluid (water) significantly enhanced heat transfer within the heat exchanger due to their superior thermophysical properties, showing consistency with the findings reported in the literature. Specifically, the heat transfer rate increased by 7.16% at a nanoparticle volume concentration of 0.25% compared with pure water. For concentrations of 0.50%, 0.75%, 1.00%, and 1.25%, the corresponding increases were 15.47%, 23.32%, 30.76%, and 37.83%, respectively.

The maximum enhancement in the heat-transfer coefficient was achieved at a nanoparticle volume concentration of 1.25%, representing a 47.84% increase compared to that of pure water. Conversely, the highest pressure drop also occurred at the 1.25% concentration, resulting in a 5.30% penalty relative to that of the base fluid.

The generated temperature profiles provided valuable information about the effects of nanofluid properties on thermal behavior. According to the temperature profiles obtained from the cross-sectional contours of the shell, a uniform heating is observed from 0.015 m onward for both water and the nanofluid. However, these profiles indicate that the average temperature of the nanofluid is higher than that of water, which is attributed to its higher thermal conductivity and lower specific heat capacity, which facilitate greater energy absorption. This behavior is also evidenced by comparing the temperature profiles along the heat-exchanger length for different nanoparticle concentrations. It was observed that the nanofluid produced an outlet temperature higher than that of water, depending on the nanoparticle concentration, thereby enhancing the overall heat transfer performance.

It was observed that the highest pressure drop takes place at the heat-exchanger inlet, resulting from the geometric constraints of the inlet section. Furthermore, the number and configuration of the baffles contribute significantly to the total pressure drop because of the induced recirculation zones. For this reason, future studies could explore the impact of various geometric modifications as strategies to reduce the associated pressure drop. It was also verified that the pressure drop increases accordingly with increasing mass flow rate and nanoparticle concentration. This behavior is attributed to the intensification of turbulence driven by higher flow rates and the increase in fluid viscosity caused by the presence of the nanoparticles.

In addition, a regression model based on the CFD simulation results was proposed. It was observed that the R-squared value of the resulting model is 0.9998, indicating a high level of accuracy and reliability for the proposed regression under the geometry adopted in this study.

From an engineering perspective, the results demonstrated that dilute Al_2_O_3_/water nanofluids can enhance shell-side thermal performance while maintaining relatively low pressure drop and pumping power penalties. From a practical design perspective, low nanoparticle concentrations may therefore represent a more feasible operating condition for shell-and-tube heat exchangers than higher concentrations, which are commonly associated with an excessive viscosity increase and hydraulic resistance. The main contribution of this work lies in the combined evaluation of heat-transfer enhancement, pressure drop, and pumping power, demonstrating that low-concentration Al_2_O_3_/water nanofluids provide a favorable balance between thermal performance and hydraulic penalties under shell-side operating conditions. This integrated thermohydraulic assessment offers a more practical basis for the design and optimization of shell-and-tube heat exchangers than heat-transfer analysis alone. Future studies should extend the present work through experimental validation, entropy-generation analysis based on the second law of thermodynamics, the evaluation of alternative heat-exchanger geometries and hybrid nanofluids, and the assessment of industrial operating conditions to further identify optimal thermohydraulic performance.

## Nomenclature

A_o_: heat transfer area of the tubes, m^2^
c_p_: specific heat, J/kg °C
C_1_: model constant
C_2_: model constant
C_3_: minimum concentration of nanoparticles
*C*_*ε*_1__: model constant
d_o_: tube diameter, m
f_drag_: friction factor
*g⃗*: acceleration of gravity, m·s^–2^
H: enthalpy, kJ/kg
k: thermal conductivity, W/m °C
L_t_: tube length, m
m: mass, kg
*ṁ*: mass flow rate, kg·s^–1^
m_3_: minimum mass flow rate, kg·s^–1^
N_t_: number of tubes
T: temperature, °C
p: pressure, kPa
P_pp_: pumping power, W
*Q̇*: heat transfer rate, kW
Q_3_: minimum heat transfer rate, kW
q_m_+q_t_: increased energy diffusion due to turbulence, W·m^–2^
Re: Reynolds number
S_ij_: time-averaged strain rate of the main flow, s^–1^
U_o_: heat transfer coefficient, kW/m^2^ °C
*u⃗*: velocity field, m·s^–1^
*u̅*_*i*_: component i of the velocity vector, m·s^–1^
*u̅*_*j*_: component j of the velocity vector, m·s^–1^
*u̅*_*pq*_: relative velocity, m·s^–1^
*u̅*_*dr,p*_: drift velocity, m·s^–1^
x_1_: mass flow rate representation
x_2_: representation of nanoparticles concentration
y: heat transfer rate representation
**Greek symbols**
ρ: Density, kg/m^3^
μ: dynamic viscosity, kg/(m·s)
Φ: viscous dissipation, W·m^–3^
τ_*ij*_^’^: Reynolds stress tensor, kg/(m·s^2^)
κ: turbulent kinetic energy, m^2^·s^–2^
*ε*: rate of dissipation of turbulent kinetic energy, m^2^·s^–2^
η: ratio of turbulent time scale to the time-averaged strain rate
φ: volume fraction
μ_*t*_: turbulent viscosity, kg/(m·s)
*∇⃗*: vector operator, m^–1^
τ: viscous tensor, Pa
μ_*m*_ + μ_*t*_: increased energy diffusion due to turbulence, kg/(m·s)
*ΔT*_*ml*_: logarithmic mean temperature difference, °C
Δ*p*: pressure drop
**Subscripts**
k: phase k
m: mixture
p: secondary phase
q: primary phase
np: nanoparticles
fb: base fluid
nf: nanofluid
**Abbreviations**
CFD: computational fluid dynamics
DNS: direct numerical simulation
LES: large eddy simulation
RANS: reynolds averaged navier-stokes equations
RSM: response surface methodology
